# Functional impact of the head domain variants of *DES* (Desmin) on filament assembly

**DOI:** 10.1016/j.gendis.2024.101238

**Published:** 2024-02-02

**Authors:** Sabrina Voß, Volker Walhorn, Stephanie Holler, Anna Gärtner, Greta Pohl, Jens Tiesmeier, Jan Gummert, Dario Anselmetti, Hendrik Milting, Andreas Brodehl

**Affiliations:** a Erich and Hanna Klessmann Institute, Heart and Diabetes Center NRW, University Hospital of the Ruhr-University Bochum, Georgstrasse 11, Bad Oeynhausen D-32545, Germany; b Experimental Biophysics & Applied Nanoscience, Faculty of Physics, Bielefeld University, Universitätsstrasse 25, Bielefeld 33615, Germany; c Institute for Anesthesiology, Intensive Care- and Emergency Medicine, MKK-Hospital Luebbecke, Campus OWL, Ruhr-University Bochum, Virchowstrasse 65, Lübbecke 32312, Germany

Desmin is a muscle-specific intermediate filament protein, which plays a significant role in providing structural integrity of cardiomyocytes by connecting different cell organelles and multi-protein complexes.^1^ Pathogenic *DES* mutations cause different cardiomyopathies and skeletal myopathies.^2^ The most obvious hallmark of pathogenic *DES* mutations is an aberrant cytoplasmic desmin accumulation.^3^ However, driven by a broad clinical application of next-generation sequencing techniques and advanced classification criteria, the number of variants of unknown significance increased significantly during the last years. Especially, the impact of *DES* variants within the N-terminal head domain on the filament assembly is widely unknown.

Therefore, we generated a set of 85 different uncharacterized variants of unknown significance listed in genetic disease databases (https://www.ncbi.nlm.nih.gov/clinvar/, https://www.hgmd.cf.ac.uk/), which are localized in the desmin head domain. In addition, we created a set of eleven N-terminal deletion mutations of different sizes. The effects on filament formation were analyzed in cell culture by confocal microscopy using SW-13 cells without endogenous desmin expression (Fig. 1A; Fig. S1, 2), cardiac H9c2 myoblasts (Fig. 1B; Fig. S3), and cardiomyocytes derived from induced pluripotent stem cells (Fig. 1C; Fig. S4). These experiments indicated that the majority of missense mutations in the head domain did not interfere with desmin filament assembly (Fig. S1, 2). However, the desmin variants (p.S13P, -p.N107D, -p.E108G, and -p.K109E) inhibited the filament assembly significantly leading to aberrant cytoplasmic desmin aggregates (Fig. 1A–C). Next, we expressed and purified wild-type and mutant recombinant desmin (Fig. S5, 6) and analyzed *in vitro* the filament assembly by atomic force microscopy (Fig. 1D). Wild-type desmin formed filaments of different lengths. In contrast, desmin-p.S13P formed only small structures, which are presumably unit-length filaments based on their size. The mutants p.N107D and p.K109E formed larger filamentous aggregates. Desmin-p.E108G formed *in vitro* small fibrils and filaments with sharp kinks and edges. These experiments revealed for all four desmin mutants aberrant desmin structures indicating intrinsic filament assembly defects (Fig. 1D).

**Figure 1:**
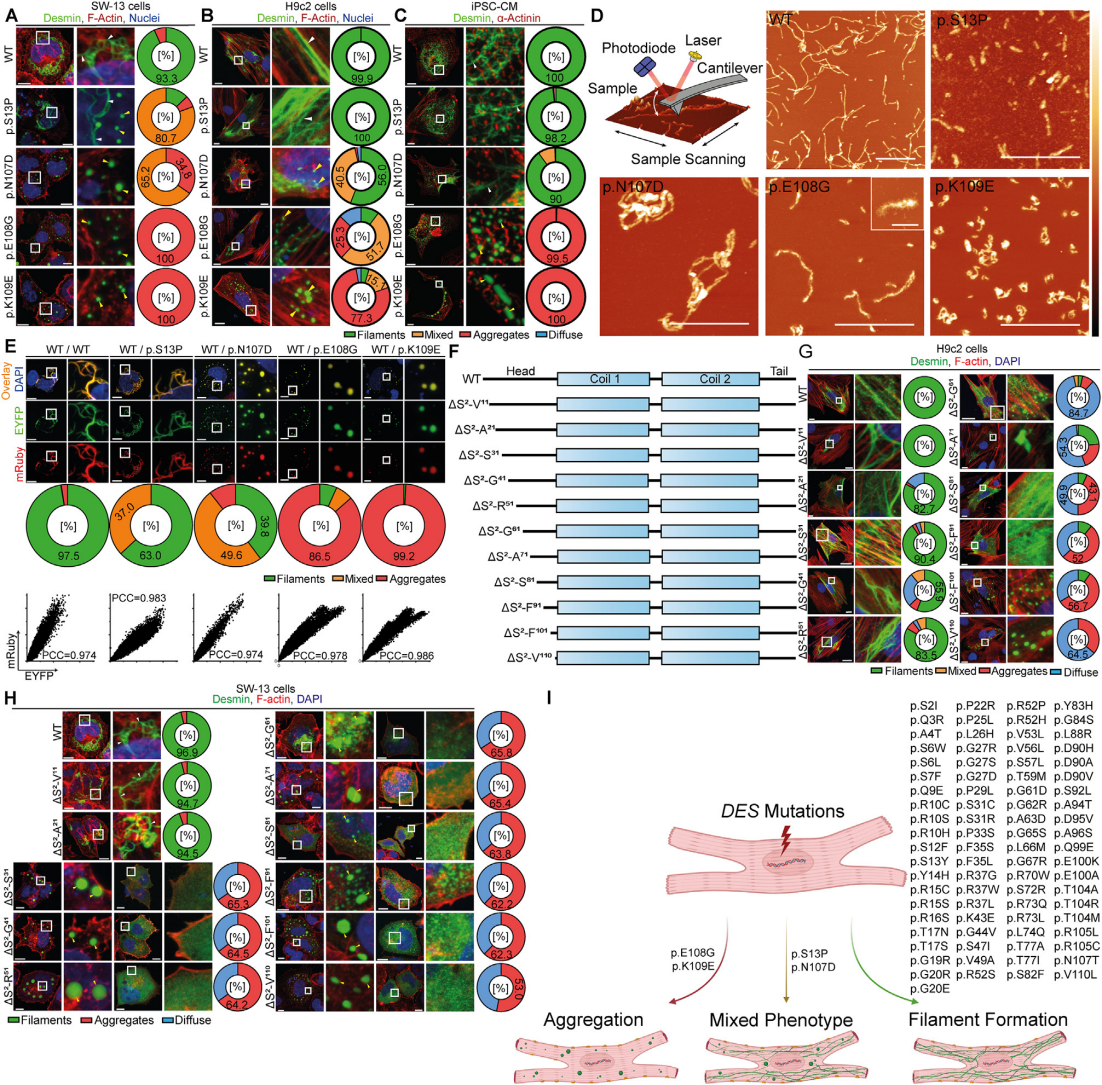
Functional analysis of *DES* variants of unknown significance localized in the desmin head domain. **(A)** Representative maximal intensity projections of SW-13 cells expressing wild-type or mutant desmin (shown in green). F-actin is shown in red and the nuclei are shown in blue. Scale bars represent 10 μm. The percentages of the different cell phenotypes (filaments, mixed, aggregates or diffuse localization) are summarized as pie charts. Wild-type desmin forms in the majority of transfected SW-13 cells (93.3 %) filamentous structures (white arrows). Of note, two desmin mutants (p.S13P and p.N107D) form in most cells mixed phenotypes and two other desmin mutants (p.E108G and p.K109E) form predominant cytoplasmic aggregates (yellow arrows). **(B)** Representative maximal intensity projections of H9c2 cells expressing wild-type or mutant desmin (shown in green). F-actin and the nuclei are shown in red or blue. Scale bars represent 20 μm. The percentages of different cell phenotypes (filaments, mixed, aggregates or diffuse localization) are summarized as pie charts. Desmin-WT, -p.S13P and -p.N107D form in the majority of transfected H9c2 cells filamentous structures. Of note, the desmin mutants (p.E108G and p.K109E) form predominantly cytoplasmic aggregates or a mixed phenotype (yellow arrows). **(C)** Representative maximal intensity projections of cardiomyocytes derived from induced pluripotent stem cells expressing wild-type or mutant desmin (shown in green). α-Actinin, as a cardiomyocyte marker, is shown in red. Scale bars represent 20 μm. The percentages of different cell phenotypes (filaments, mixed, aggregates or diffuse localization) are summarized as pie charts. Desmin-WT, -p.S13P and -p.N107D form in the majority of transfected H9c2 cells filamentous structures. The desmin mutants (p.E108G and p.K109E) form predominantly cytoplasmic aggregates (yellow arrows). **(D)** Working principle of an atomic force microscopy and *in vitro* filament assembly of recombinant mutant and wild-type desmin. Typical topographical atomic force microscopy scans of recombinant desmin after assembly are shown. Wild-type desmin exhibits long (several 100 nm) predominantly straight (stiff) filaments. The filament assembly of desmin-p.S13P appears to be severely impaired. Only short fibrils with lengths from about 50 to a few hundred nanometers were observed. Desmin-p.N107D shows coiled filaments and filamentous aggregates with a length of up to a few micrometers. The small filament curvature suggests a rather low persistence length. Desmin-p.E108G exhibits short filaments and protofilaments. The fringed hem of the filaments indicates that desmin monomers/oligomers are either only partially incorporated into the filament or that the filaments are inherently unstable and easily decompose into their constituents. Desmin-p.K109E shows short predominantly curly filamentous aggregates and protofilaments. The scale bars correspond to 1 μm or 200 nm (inset) whereas the color bar corresponds to a height of 20 nm (WT, p.S13P, p.E108G), 30 nm (p.K109E), or 40 nm (p.N107D), respectively. **(E)** Co-expression experiments of mutant and wild-type desmin in SW-13 cells. Representative images of double-transfected SW-13 cells, expressing wild-type desmin (fused to mRuby, red) and wild-type or mutant desmin (fused to EYFP, green) are shown. The overlay is shown in yellow. Nuclei were stained using DAPI (blue). Scale bars represent 10 μm. Representative scatter plots of the mRuby and EYFP channels are shown. The percentages of the different cell phenotypes are summarized as pie charts. **(F)** Schematic overview of the generated N-terminal desmin deletion mutations. **(G)** Representative images of H9c2 cells expressing wild-type desmin or the deletion mutants are shown. Desmin is shown in green, F-actin in red, and the nuclei in blue. Scale bars represent 20 μm. Pie charts of observed subcellular desmin localization are shown. **(H)** Representative images of SW-13 cells expressing wild-type desmin or the deletion mutants are shown. In case of aggregate and diffuse localization, two typical cell images are shown. Desmin is shown in green, F-actin in red, and the nuclei in blue. The yellow arrowheads indicate cytoplasmic aggregates and the white arrowheads indicate filamentous structures. Scale bars represent 10 μm. Pie charts summarizing the subcellular desmin localization are shown. **(I)** Atlas of desmin (*DES*) mutations. Impact of *DES* mutations within the non-helical head domain on filament/aggregate formation.

Most of the known *DES* mutation carriers present a heterozygous genotype. Subsequently, we performed co-transfection experiments using mutant and wild-type desmin fused to different fluorescent proteins to model a heterozygous status (Fig. S7). In the case of desmin-p.S13P, the majority of double-transfected SW-13 cells showed filaments comparable to the wild-type double-transfected cells (Fig. 1E; Fig. S8). The co-localization analysis determining the Pearson correlation coefficients revealed that wild-type and desmin-p.S13P were incorporated into the same filaments similar to the wild-type/wild-type control experiment (Fig. 1E; Fig. S9). In the majority of transfected SW-13 cells, desmin-p.N107D formed in the presence of wild-type desmin a mixture of filaments and aberrant cytoplasmic aggregates consisting of both desmin forms (Fig. 1E; Fig. S8). In contrast, desmin-p.E108G and -p.K109E dominantly inhibited the filament assembly if they were co-expressed together with wild-type desmin. In H9c2 cells, we found similar effects of the investigated mutants, if co-expressed together with wild-type desmin (Fig. S10). These findings might explain the dominant negative inheritance of desminopathies frequently observed in cardiovascular genetics. Of note, three of the conspicuous variants are localized at the C-terminus of the head domain close to the 1A-subdomain, which we recognized recently as a genetic hot spot for cardiomyopathies^4^ indicating that this hot spot is extended to the head domain.

Since it is known that the N-terminal head domain of desmin is necessary for the regular filament assembly,^5^ we determined systematically which parts of the head domain are necessary for filament formation by generation and analysis of eleven different deletions of different sizes (Fig. 1F). Desmin deletion mutants missing 10–50 amino acids behind the first methionine were incorporated into filaments in the majority of transfected H9c2 cells (Fig. 1G; Fig. S11). However, in SW-13 cells, only desmin mutants missing the first 10–20 amino acids formed intermediate filaments comparable to wild-type desmin (Fig. 1H; Fig. S12). All other larger N-terminal deletions caused a cytoplasmic aggregation or diffuse desmin localization in transfected cells (Fig. 1G, H; Fig. S11, 12) demonstrating that the size of the N-terminal head domain of desmin is highly relevant for filament assembly.

In conclusion, our study provides functional data about variants within the head domain of desmin and can be used in cardiovascular genetics as an “atlas*”* of *DES* variants contributing to functional variant classification in the future (Fig. 1I).

## Author contributions

Conceptualization: A.B.; Data curation: S.V., V.W., S.H., and A.B.; Formal analysis: S.V., V.W., and A.B.; Funding acquisition: A.B. and H.M.; Investigations: S.V., V.W., S.H., and A.B.; Project administration: A.B.; Resources: A.G., J.G., D.A., and H.M.; Supervision: A.B.; Validation: S.V., V.W., and A.B.; Visualization: S.V., V.W., and A.B.; Roles/Writing - original draft: A.B.; Writing - review & editing: S.V., V.W., S.H., A.G., G.P., J.T., J.G., D.A., and H.M..

## Conflict of interests

A.B. is a shareholder of Tenaya Therapeutics, Bayer and Prime Medicine. All other authors do not have any conflict of interest.

## Funding

A.B. and H.M. are thankful for the financial support of the Ruhr-University Bochum (FoRUM, F1074-2023) and of the Deutsche Herzstiftung e.V. (Frankfurt a.M.). We acknowledge support by the Open Access Publication Funds of the Ruhr-Universität Bochum.
