# Supplementary material for: Functional impact of the head domain variants of DES (Desmin) on filament assembly
Source: Genes Dis. 2024 Feb 2;12(1):101238. doi: 10.1016/j.gendis.2024.101238 (PMC11620975; doi:10.1016/j.gendis.2024.101238)
Supplement: Multimedia component 1 [file mmc1.docx]

**Supplementary – Material and Methods**

to

Atlas of *DES* (desmin) variants: Impact of variants located within the head domain on filament assembly

**Genetic Disease Database Analysis**

85 rare missense VUS affecting amino acids within the desmin head domain were selected from the Human Gene Mutation Database (<https://www.hgmd.cf.ac.uk/>) and the ClinVar Database (<https://www.ncbi.nlm.nih.gov/clinvar/>, accessed on 10^th^ January 2023). A minor allele frequency (MAF) <0.0001 (Genome Aggregation Database, <https://gnomad.broadinstitute.org/>) was used as an exclusion criterion since it is above the prevalence of cardiomyopathies.

**Cloning and Site-Directed Mutagenesis**

The plasmid pEYFP-N1-DES-WT was generated by ligation of the human *DES* cDNA via *Xho*I and *BamH*I restriction sites as previously described [1] (Fig. S13A). Cloning of pmRuby-N1-DES-WT was previously described [2] (Fig. S13B). The bacterial expression plasmid pET100D-TOPO-DES was generated by TOPO cloning according to the manufacturer’s instructions (Thermo Fisher Scientific, Waltham, MA, USA) and was previously described [1] (Fig. S13C). Most missense and deletion mutations were inserted by site-directed mutagenesis using the QuikChange Lightning Kit (Agilent Technologies, Santa Clara, CA, USA) in combination with appropriate oligonucleotides (Table S1, synthesized by Microsynth, Balgach, Switzerland) according to the manufacturer’s instructions. *Escherichia coli* (*E. coli*, XL-10 gold) bacteria were transformed by heat shock (30 sec, 42 °C) and colonies were selected overnight using 50 µg/mL kanamycin sulfate (Sigma-Aldrich, Darmstadt, Germany). Some of the N-terminal deletion mutations were generated by PCR using Phusion Polymerase (Thermo Fisher Scientific) followed by ligation using T4 DNA ligase (Thermo Fisher Scientific) (Table S1). Plasmids were prepared from single colonies using the GeneJET Plasmid Miniprep Kit (Thermo Fisher Scientific) as described by the manufacturer. All plasmids were verified in the desmin coding region by Sanger sequencing (Macrogen, Amsterdam, Netherlands) using the CMV-for and EGFP-rev primers for the pEYFP-N1-DES constructs or the T7-for and T7-rev primers for the pET100D-TOPO-DES constructs (Table S1). The sequencing data were analyzed using SnapGene 6.1 software (GSL Biotech, San Diego, CA, USA).

**Cell Culture**

Dulbecco’s Modified Eagle Medium (DMEM, Thermo Fisher Scientific) was used for culturing of H9c2 and SW-13 cells (ATCC, Manassas, VA, USA) at 37 °C and 5 % CO_2_. DMEM was supplemented with 10 % fetal calf serum and penicillin/streptomycin. Cells were sub-cultured at a confluency of >90 % using trypsin/ethylendiaminetetraacetic acid (Thermo Fisher Scientific).

Human induced pluripotent stem cells (iPSCs) generated from a healthy donor (NP0040‑8, UKKi011-A, kindly provided by Dr. Tomo Saric, University of Cologne, Germany) were cultured in Essential 8 Medium (Thermo Fisher Scientific) on vitronectin-coated plates. Versene solution (Thermo Fisher Scientific) was used for cell dissociation.

**Cardiomyocyte Differentiation of Induced Pluripotent Stem Cells**

iPSCs were differentiated into cardiomyocytes by modulation of the Wnt-pathway as previously described [3].

**Cell Transfection**

24 h before transient transfection, SW-13 and H9c2 cells were subcultured and 200.000 cells were cultured in 8‑well µSlide chambers (ibidi, Gräfelfing, Germany) or in 6-well plates for expression analysis. Beating iPSC-derived cardiomyocytes were split using Accutase / trypsin (1:1; Sigma-Aldrich) for 6 min at 37 °C, centrifuged and cultured on vitronectin-coated (Sigma-Aldrich) µSlide chambers. The cells were transiently transfected using Lipofectamin 3000 (Thermo Fisher Scientific) according to the manufacturer’s instructions. Afterwards, the cells were cultured for 24 h at 37 °C and 5 % CO_2_.

**Cell Fixation and Immunocytochemistry**

Cells were washed with phosphate buffered saline (PBS, Thermo Fisher Scientific). 4 % HistoFix (Carl Roth, Karlsruhe, Germany) was used for cell fixation (15 min at room temperature, RT). After two washing steps with PBS, the cells were permeabilized using 0.1 % Triton X-100 (15 min, RT). Subsequently, the cells were washed with PBS. Phalloidin conjugated with the fluorescence dye Texas Red (1:400, Thermo Fisher Scientific) was used for staining of F-actin (40 min, RT), 4′,6-diamidino-2-phenylindole (DAPI, 1 µg/mL) was used for staining of the nuclei (5 min, RT). α-Actinin was stained by using antibodies as recently described [4]. Finally, two washing steps with PBS were performed and the cells were stored at 4 °C until confocal microscopy analyses.

**Confocal Microscopy**

Confocal laser scanning microscopy was performed using the TCS SP8 system (Leica Microsystems, Wetzlar, Germany) as previously described [5]. Briefly, DAPI, EYFP and Texas Red were excited at 405, 488 and 552 nm. The fluorescence emissions of these dyes were sequentially detected between 410-460, 493-560 and 570-758 nm. The fluorescent protein mRuby [6] was excited at 552 nm and the fluorescence emission was detected between 557-643 nm. 3D stacks were recorded and processed using the Las X software (Leica Microsystems). All representative cell images are shown as maximum intensity projections.

**Co-localization Analysis**

Co-localization of desmin-EYFP and desmin-mRuby constructs was evaluated using the Fiji software in combination with the EzColocalization plugin [7]. The Pearson correlation coefficients (PCC) of >10 double transfected cells were determined.

**Expression Analysis**

Expression analysis of desmin-EYFP was done by fluorescence intensity measurements using the Infinite M1000 plate reader (Tecan, Männedorf, Switzerland) at 37 °C. Transfected cells were washed twice with PBS and were afterwards excited at 488 nm. Fluorescence emission was determined at 505 nm with a bandwidth of 20 nm. Four independent transfection experiments per construct were analyzed and normalized by subtraction of background signals of non-transfected cells (NT, Fig. S14).

**Recombinant Desmin Expression**

E. coli (BL21 Star DE3, Thermo Fisher Scientific) were transformed with wild-type or mutant pET100D-TOPO-DES expression plasmids by heat shock treatment (30 sec, 42 °C). After selection using 100 µg/mL ampicillin over night, single colonies were inoculated and expanded in Lysogeny Broth medium (Carl Roth). Desmin expression was induced by isopropyl β-D-thiogalactoside (1 mM) for 4 h under vigorous shaking at 37 °C. Afterwards, bacteria were collected by centrifugation and stored at -80 °C.

**Purification of Recombinant Desmin**

Bacteria were lysed and the inclusion bodies were prepared as previously described [1]. Afterwards, recombinant desmin was purified under denaturing conditions (8 M urea) by ion exchange chromatography (IEC) using 5 mL HiTrap DEAE-FF columns (GE Healthcare, Chicago, IL, USA) and in a second step by immobilized metal affinity chromatography (IMAC) using HisTrap FF Crude columns (Cytiva, Marlborough, MA, USA) in combination with the ÄKTApurifier system (GE Healthcare). The purified recombinant desmin was stored at -80 °C.

**Atomic Force Microscopy**

Recombinant desmin was dialyzed stepwise in buffer without urea (5 mM Tris-HCl, 1 mM dithiothreitol, pH 8.4) and diluted to a concentration of 0.3 g/L. The filament assembly was initiated by adding an equal volume of sodium chloride buffer (200 mM NaCl, 45 mM Tris-HCl, pH 7.0) and heating to 37 °C for 1 h [8]. Poly-L-ornithine solution (molecular weight 30.000-70.000, 0.01%, Sigma-Aldrich) was applied to freshly cleaved mica substrates (Plano, Wetzlar, Germany) and incubated for 5 min. Subsequently, the mica substrates were rinsed with deionized water to remove excess polypeptides. Readily assembled desmin was applied to the modified mica substrates and incubated for approximately 30 s. Unbound desmin was removed by rinsing with deionized water. AFM imaging was performed in tapping mode at RT in water using a JPK NanoWizard ULTRA Speed 2 (JPK Bruker, Berlin, Germany) and USC-F0.3-k0.3 Cantilevers (Nano World, Neuchâtel, Switzerland).

**Molecular Modelling and Sequence Alignment**

AlphaFold Multimer was used for prediction of the human dimeric desmin structure [9]. The molecular structure was visualized by PyMOL Molecular Graphics Version 2.52. (Schrödinger, New York, NY, USA). The multiple sequence alignment (Fig. S15) was performed using Clustal Omega (<https://www.ebi.ac.uk/Tools/msa/clustalo/>, accessed on 11^th^ January 2023) [10] using the following desmin reference sequences: *Homo sapiens* (NP_001918.3), *Xenopus laevis* (NP_001080177.1), *Mus musculus* (NP_034173.1), *Rattus norvegicus* (NP_071976.2), *Danio rerio* (NP_571038.2 and NP_001070920.1).

**Statistical Analysis**

In minimum, four independent transfection experiments were performed per construct and per cell line. In each transfection experiment, about 100 transiently transfected cells were analyzed. The percentage of aggregate formation was calculated for each independent experiment. The non-parametric Kruskal-Wallis test followed by Dunn’s multiple comparison was used for statistical analysis using GraphPad Prism Version 9.0 (GraphPad Software, San Diego, CA, USA). All data are shown as means ± standard deviations (SD).

**Supplementary -Material and Methods - References**

1. Brodehl, A., et al., Dual color photoactivation localization microscopy of cardiomyopathy-associated desmin mutants. J Biol Chem, 2012. 287(19): p. 16047-57.

2. Brodehl, A., et al., Functional characterization of the novel DES mutation p.L136P associated with dilated cardiomyopathy reveals a dominant filament assembly defect. J Mol Cell Cardiol, 2016. 91: p. 207-14.

3. Brodehl, A., et al., A homozygous DSC2 deletion associated with arrhythmogenic cardiomyopathy is caused by uniparental isodisomy. J Mol Cell Cardiol, 2020. 141: p. 17-29.

4. Brodehl, A., et al., The N-Terminal Part of the 1A Domain of Desmin Is a Hot Spot Region for Putative Pathogenic DES Mutations Affecting Filament Assembly. Cells, 2022. 11(23).

5. Kulikova, O., et al., The Desmin (DES) Mutation p.A337P Is Associated with Left-Ventricular Non-Compaction Cardiomyopathy. Genes (Basel), 2021. 12(1).

6. Kredel, S., et al., mRuby, a bright monomeric red fluorescent protein for labeling of subcellular structures. PLoS One, 2009. 4(2): p. e4391.

7. Stauffer, W., H. Sheng, and H.N. Lim, EzColocalization: An ImageJ plugin for visualizing and measuring colocalization in cells and organisms. Sci Rep, 2018. 8(1): p. 15764.

8. Kreplak, L. and H. Bar, Severe myopathy mutations modify the nanomechanics of desmin intermediate filaments. J Mol Biol, 2009. 385(4): p. 1043-51.

9. Evans, R., et al., Protein complex prediction with AlphaFold-Multimer. 2022: p. 2021.10. 04.463034.

10. Sievers, F., et al., Fast, scalable generation of high-quality protein multiple sequence alignments using Clustal Omega. Mol Syst Biol, 2011. 7: p. 539.
