# Supplementary material for: Functional impact of the head domain variants of DES (Desmin) on filament assembly
Source: Genes Dis. 2024 Feb 2;12(1):101238. doi: 10.1016/j.gendis.2024.101238 (PMC11620975; doi:10.1016/j.gendis.2024.101238)
Supplement: Multimedia component 2 [file mmc2.docx]

**Supplementary Figures and Tables**

to

Atlas of *DES* (desmin) variants: Impact of variants located within the head domain on filament assembly


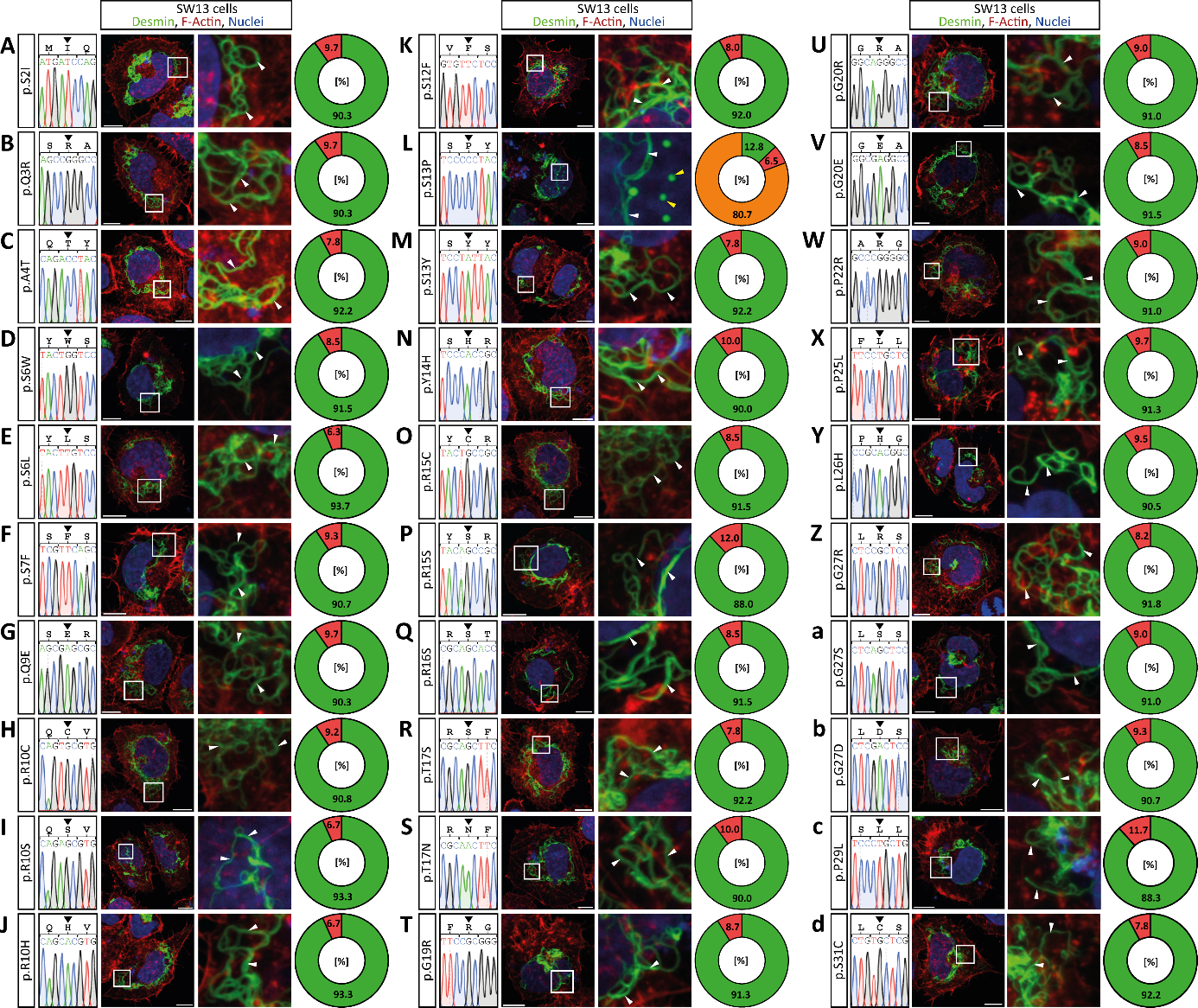


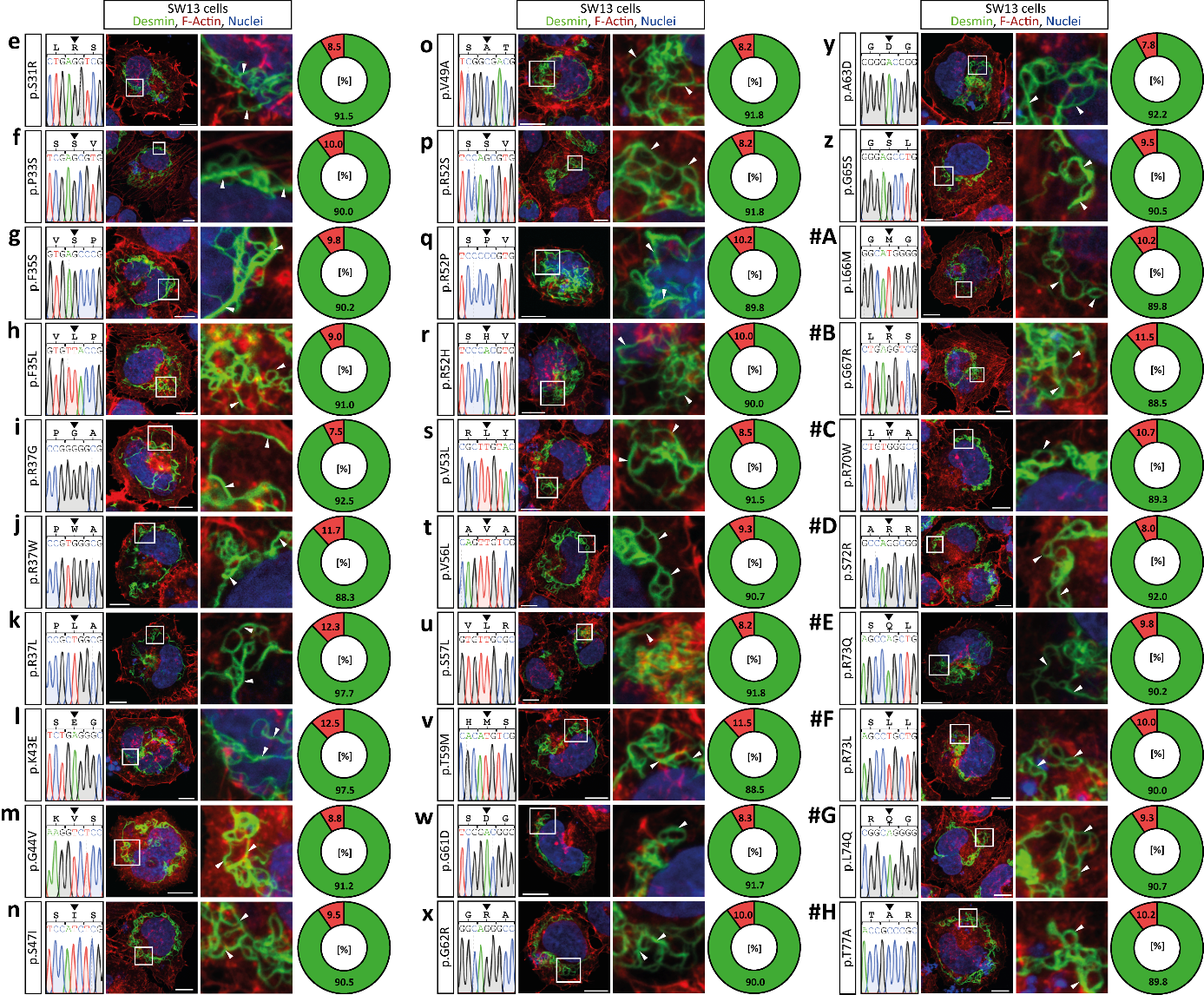


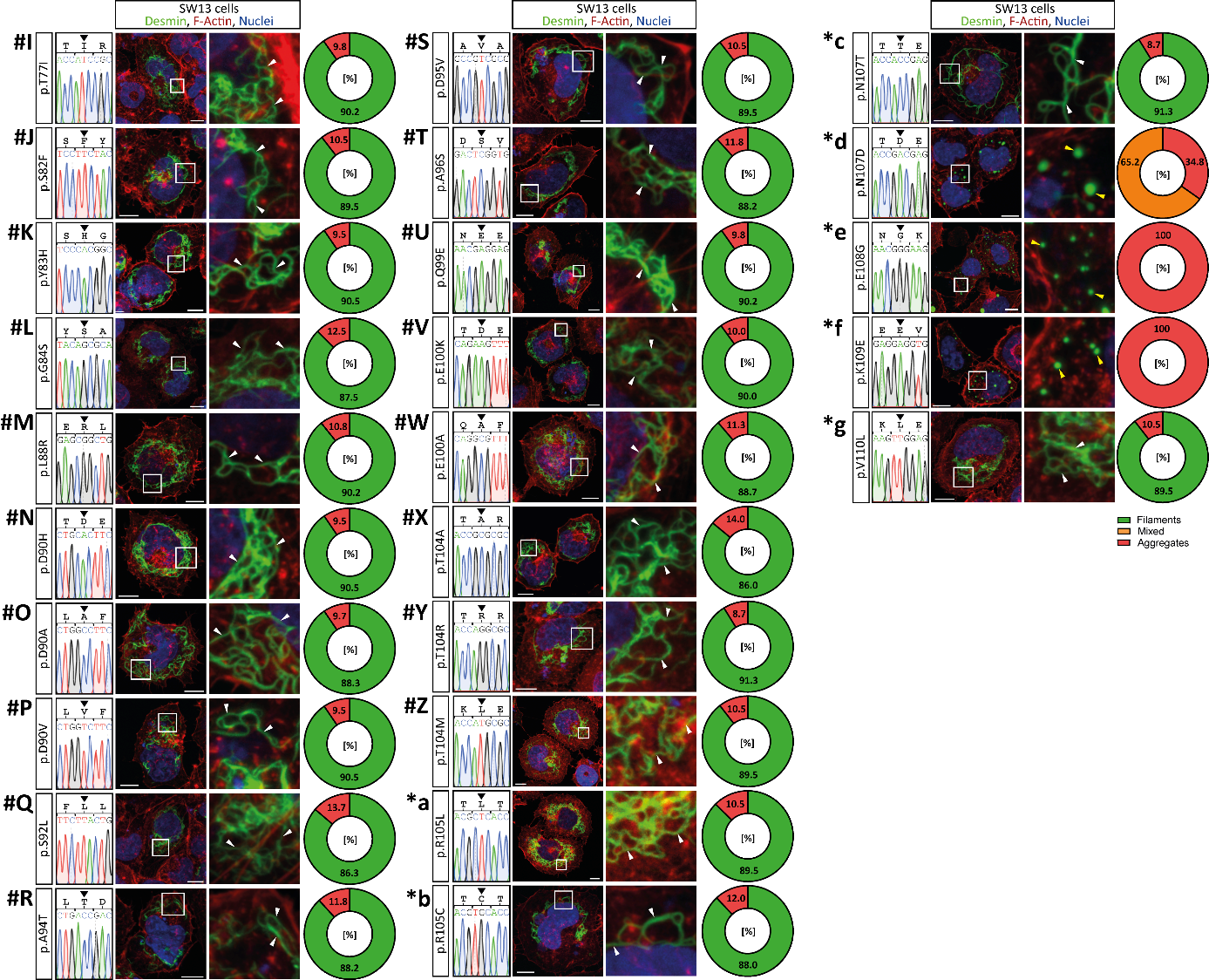


**Figure S1.** Filament formation of *DES* head domain variants in SW‑13 cells. **(A-*g)** Partial electropherograms of the 85 generated VUS constructs and representative maximal intensity projections of transfected SW-13 cells expressing mutant desmin are shown (green). F-actin was stained using phalloidin-Texas Red (red) and the nuclei were stained using DAPI (blue). Scale bars represent 10 nm. The percentage of the different cell phenotypes is summarized as pie charts. The majority of desmin mutants form filamentous structures comparable to the wild-type desmin. Of note, two desmin mutants (p.S13P and p.N107D) form in most cells mixed phenotypes **(L and *d)** and two other desmin mutants (p.E108G and p.K109E) form predominant cytoplasmic aggregates **(*e and *f)**. The yellow arrow heads indicate cytoplasmic aggregates and the white arrow heads indicate filamentous structures.


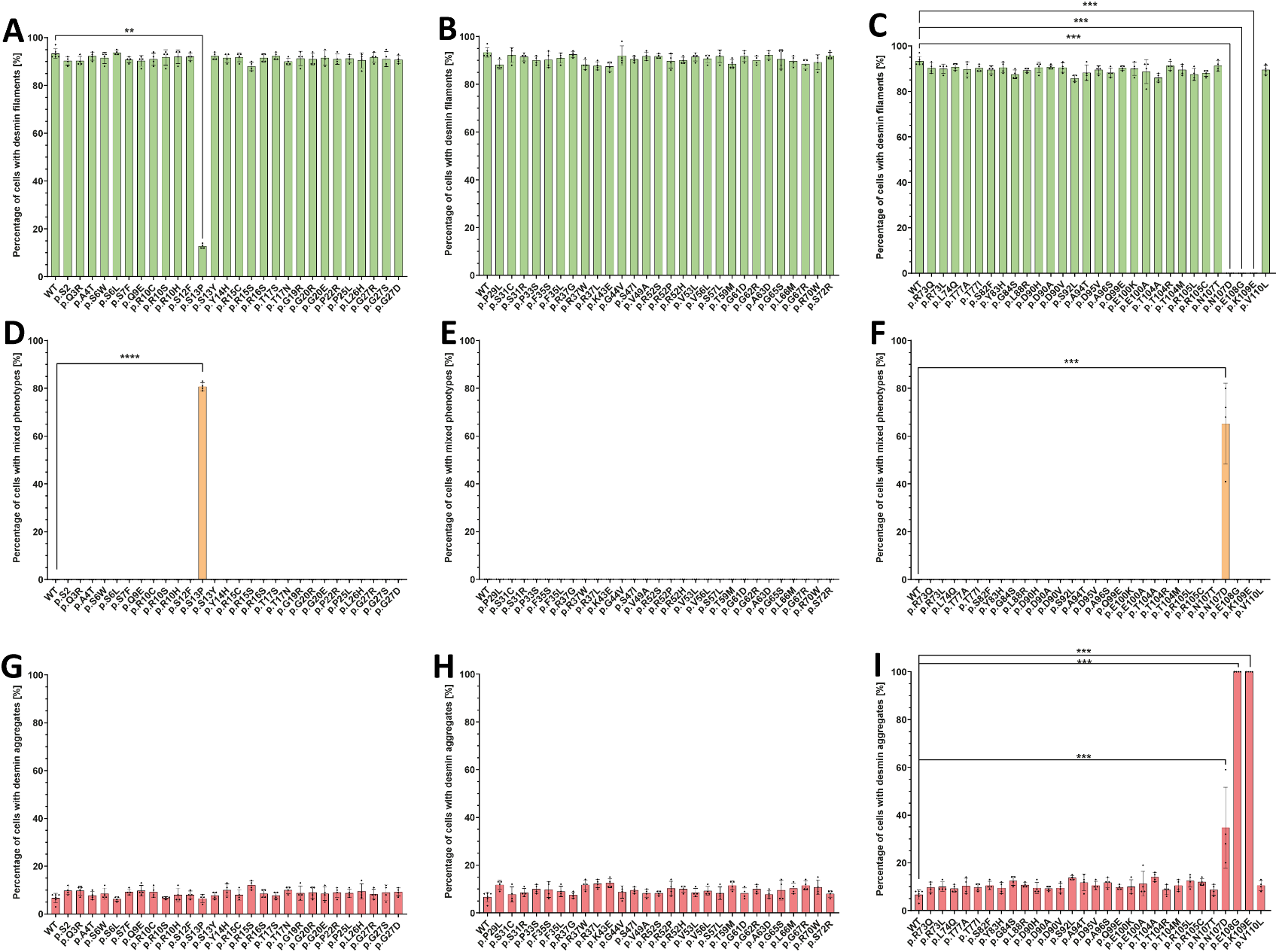


**Figure S2.** Statistical analysis of aggregate and filament formation in transfected SW-13 cells. Statistical analysis of desmin filament or aggregate formation using non-parametric Kruskal-Wallis test in transfected SW-13 cells expressing wild-type desmin or desmin deletion mutants. All data are shown as mean ± standard deviation. *p≤0.05; **p≤0.01; ***p≤0.001; ****p≤0.0001.


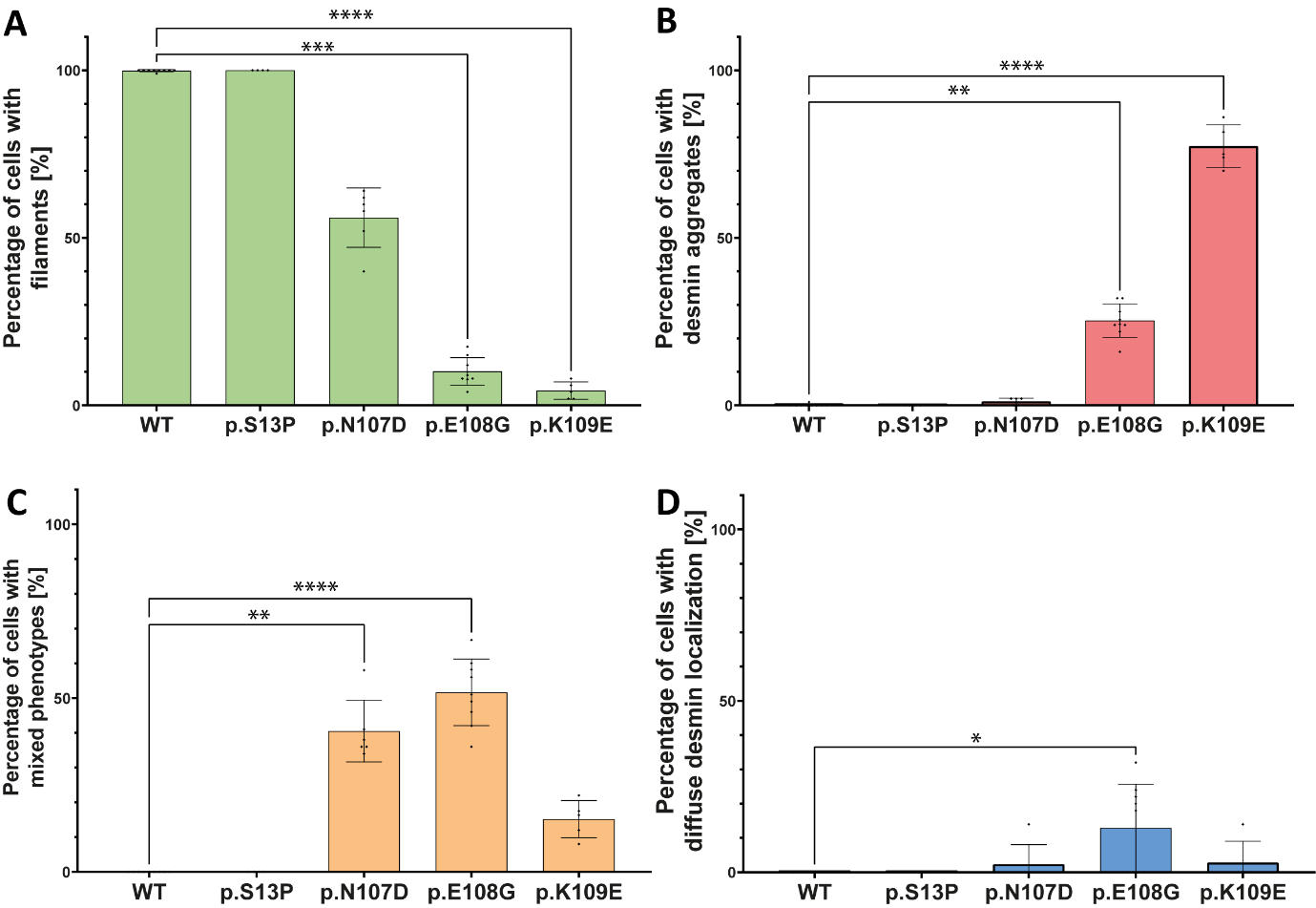


**Figure S3.** Statistical analysis of filament and aberrant aggregate formation of different desmin mutants in transfected H9c2 cells. **(A-D)** Statistical analysis was performed using non-parametric Kruskal-Wallis test. All data are shown as mean ± standard deviation. *p≤0.05; **p≤0.01; ***p≤0.001; ****p≤0.0001.


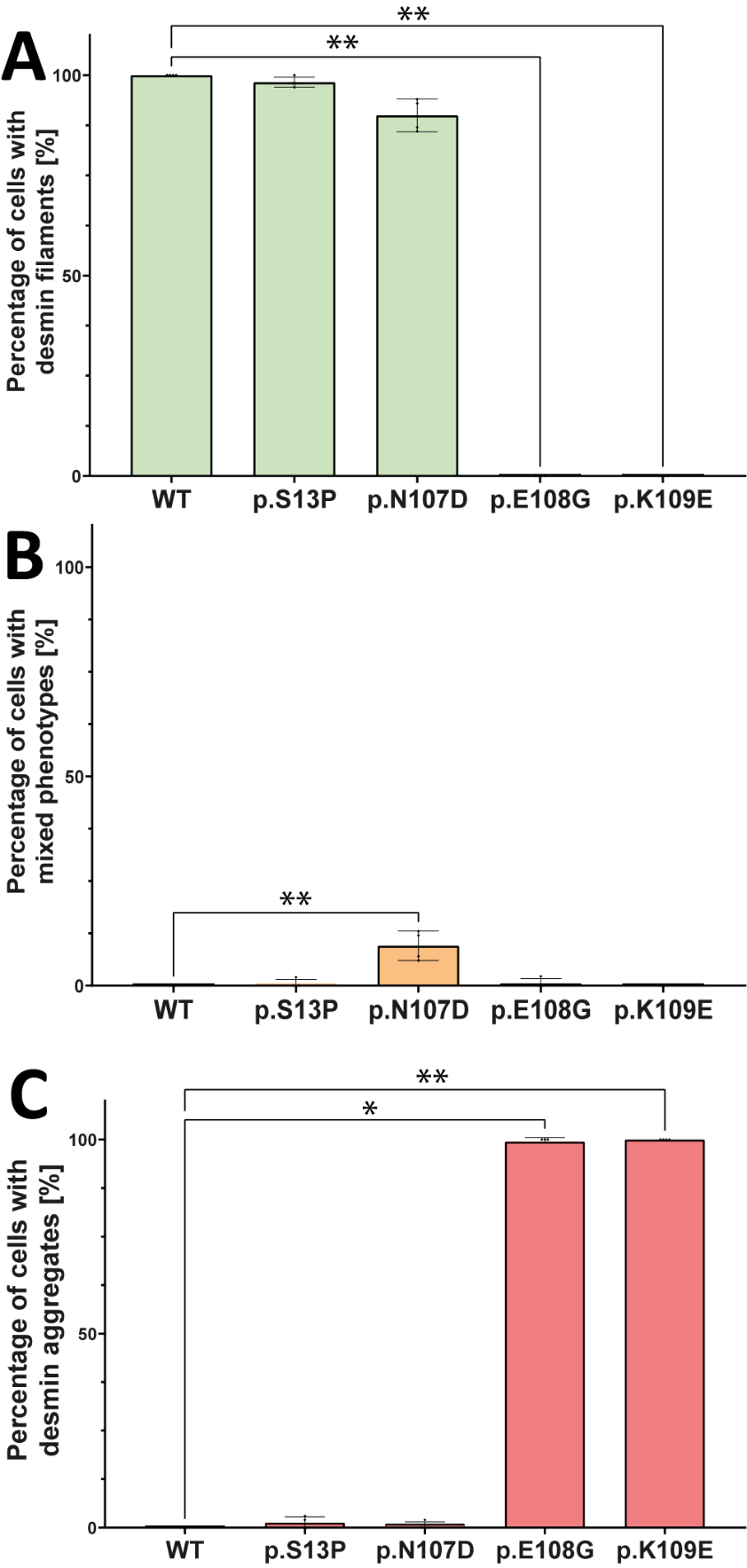


**Figure S4.** Statistical analysis of filament and aberrant aggregate formation of different desmin mutants in transfected iPSC-derived cardiomyocytes. **(A-C)** Statistical analysis was performed using non-parametric Kruskal-Wallis test. All data are shown as mean ± standard deviation. *p≤0.05; **p≤0.01.


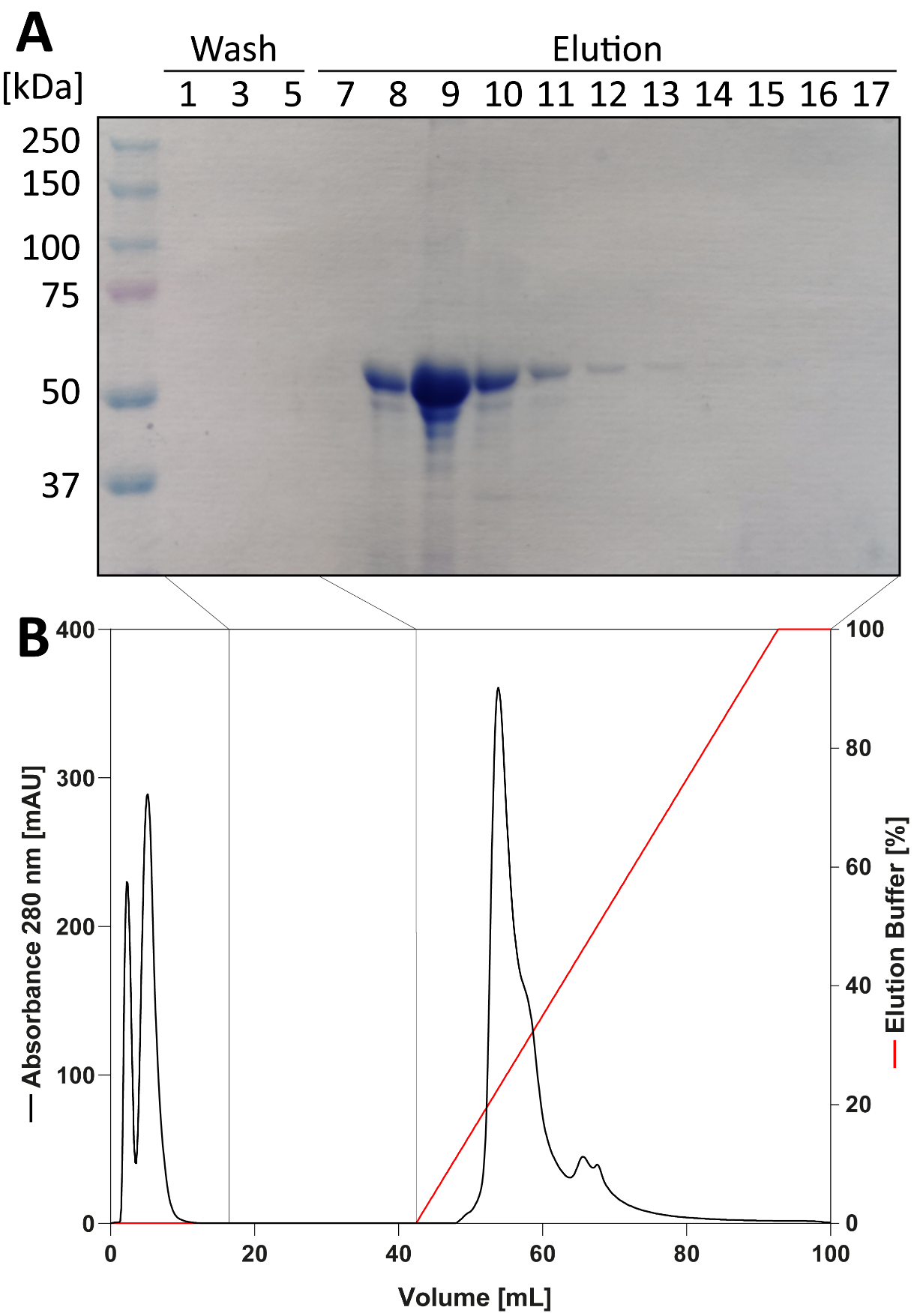


**Figure S5.** Purification of recombinant desmin by ionic exchange chromatography (IEC). **(A)** SDS-PAGE in combination with Coomassie-R250 staining of the fractions of the IEC. The elution fractions (8-10) were pooled and used afterwards for IMAC. **(B)** Chromatogram showing the elution of recombinant desmin from the IEC column. A linear gradient with increasing [NaCl] was used for elution.


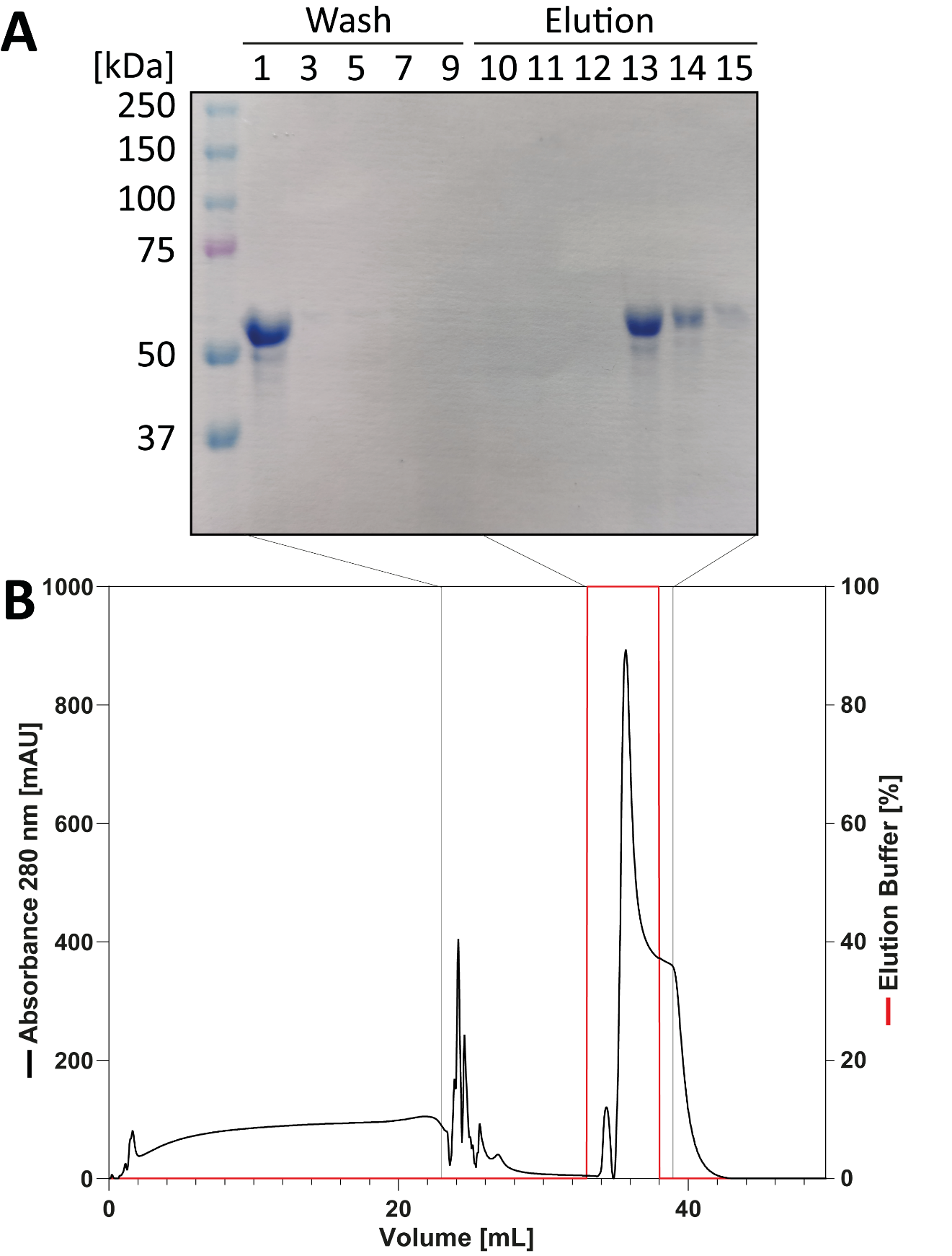


**Figure S6.** Purification of recombinant desmin by immobilized metal affinity chromatography (IMAC). **(A)** SDS-PAGE in combination with Coomassie-R250 staining of the fractions of the IMAC. **(B)** Chromatogram showing the elution of recombinant desmin from the HisTrap column. A stepwise gradient with increased imidazole concentration was used for elution.


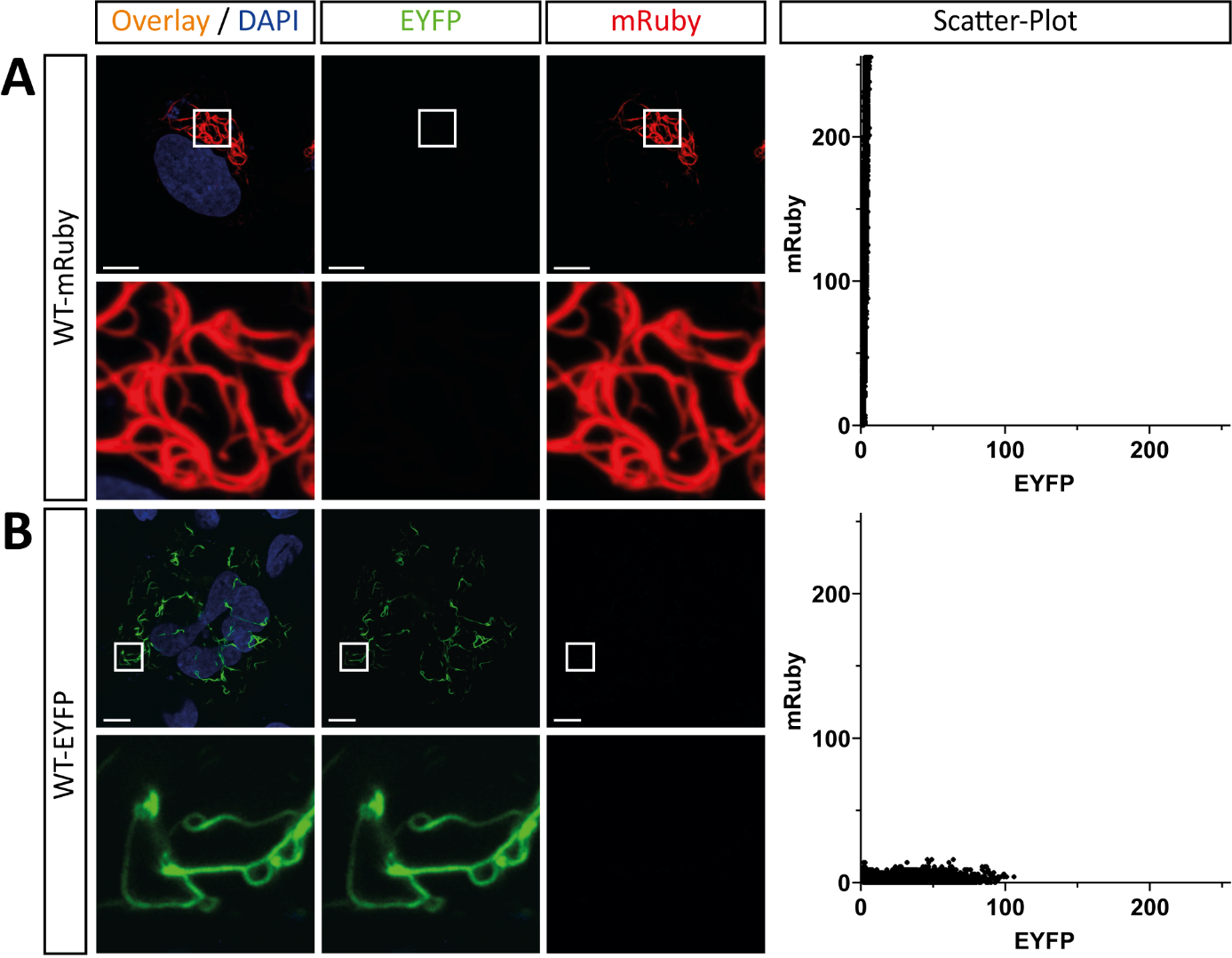


**Figure S7.** Fluorescence analysis in single transfected SW-13 cells. Representative images of single transfected cells expressing wild-type desmin fused with **(A)** mRuby or **(B)** EYFP. Scale bars represent 10 µm. Scatter plots indicate an absence of a significant crosstalk between both fluorescent proteins.


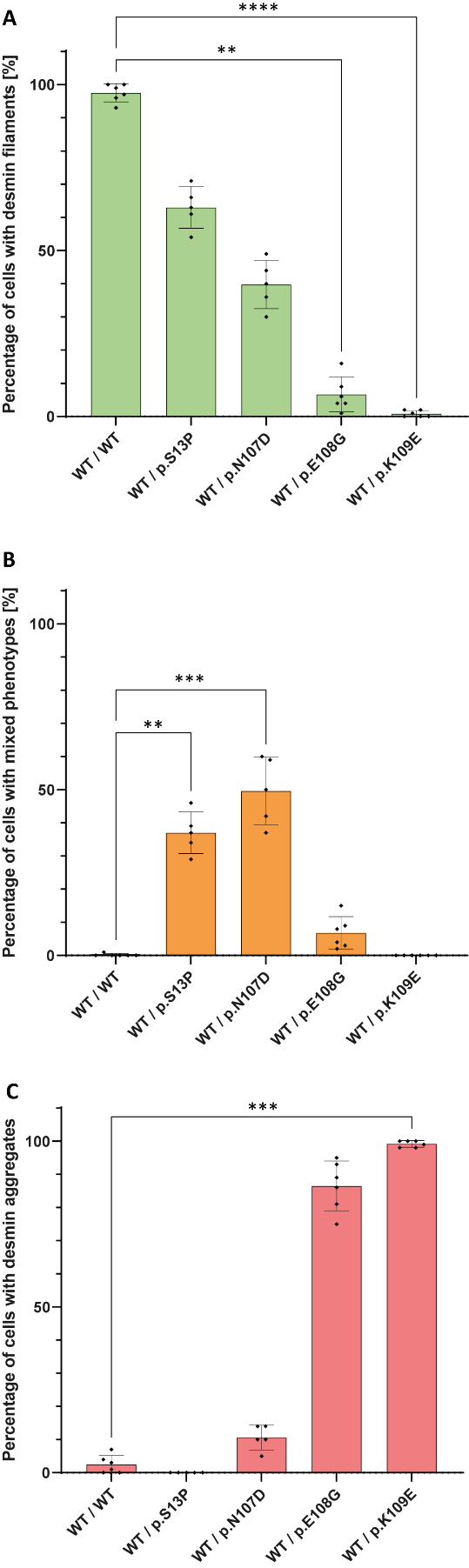


**Figure S8.** Co-expression experiments of mutant and wild-type desmin in SW-13 cells. Statistical analysis of desmin filament or aggregate formation using non-parametric Kruskal-Wallis test in double transfected SW-13 cells. All data are shown as mean ± SD. ns=not significant; *p≤0.05; **p≤0.01; ***p≤0.001; ****p≤0.0001.


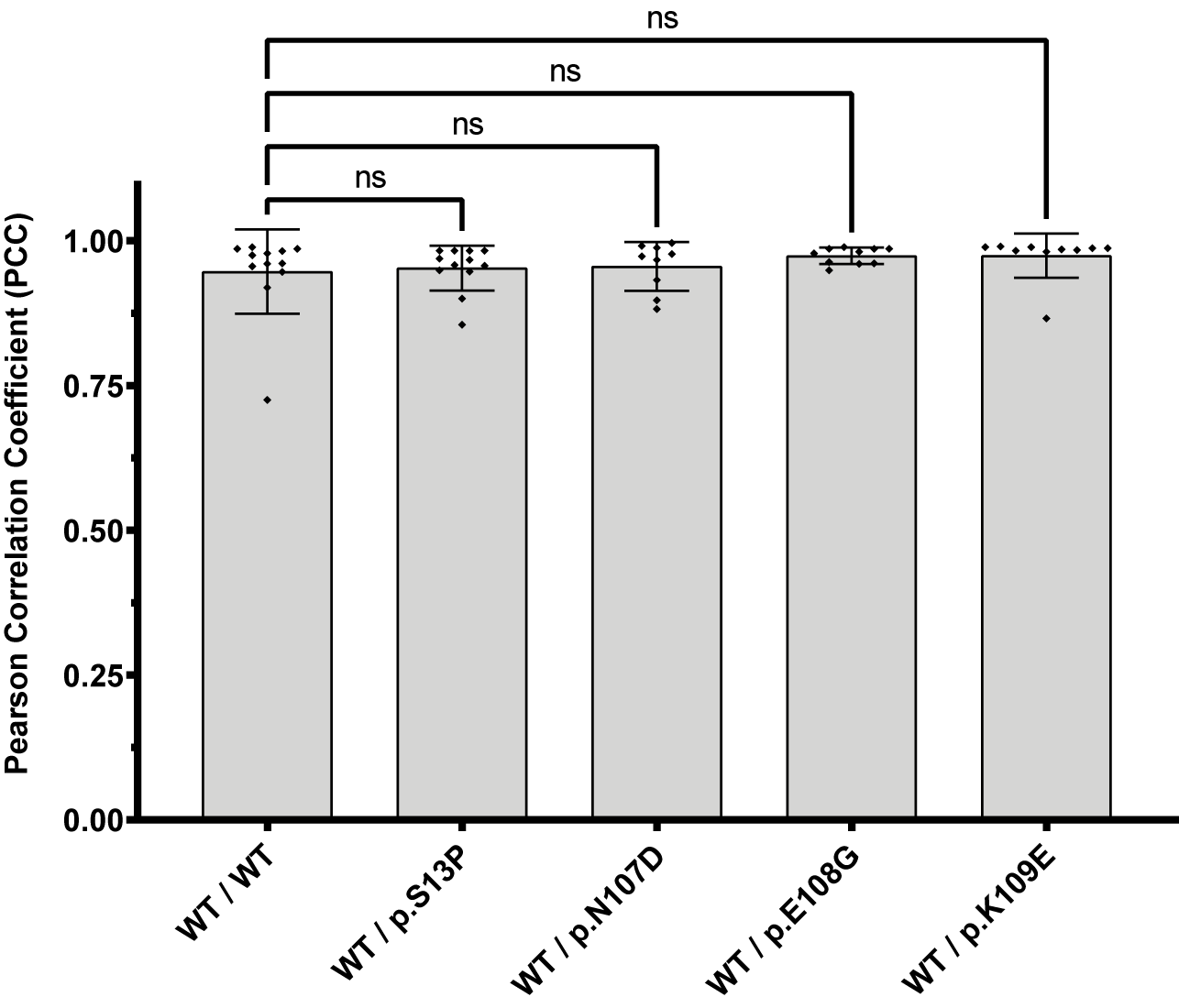


**Figure S9.** Statistical analysis of Pearson correlation coefficients of the double transfected SW-13 cells indicate a high colocalization of mutant and wild-type in filaments or aggregates. All data are shown as mean ± standard deviation. ns=not significant.


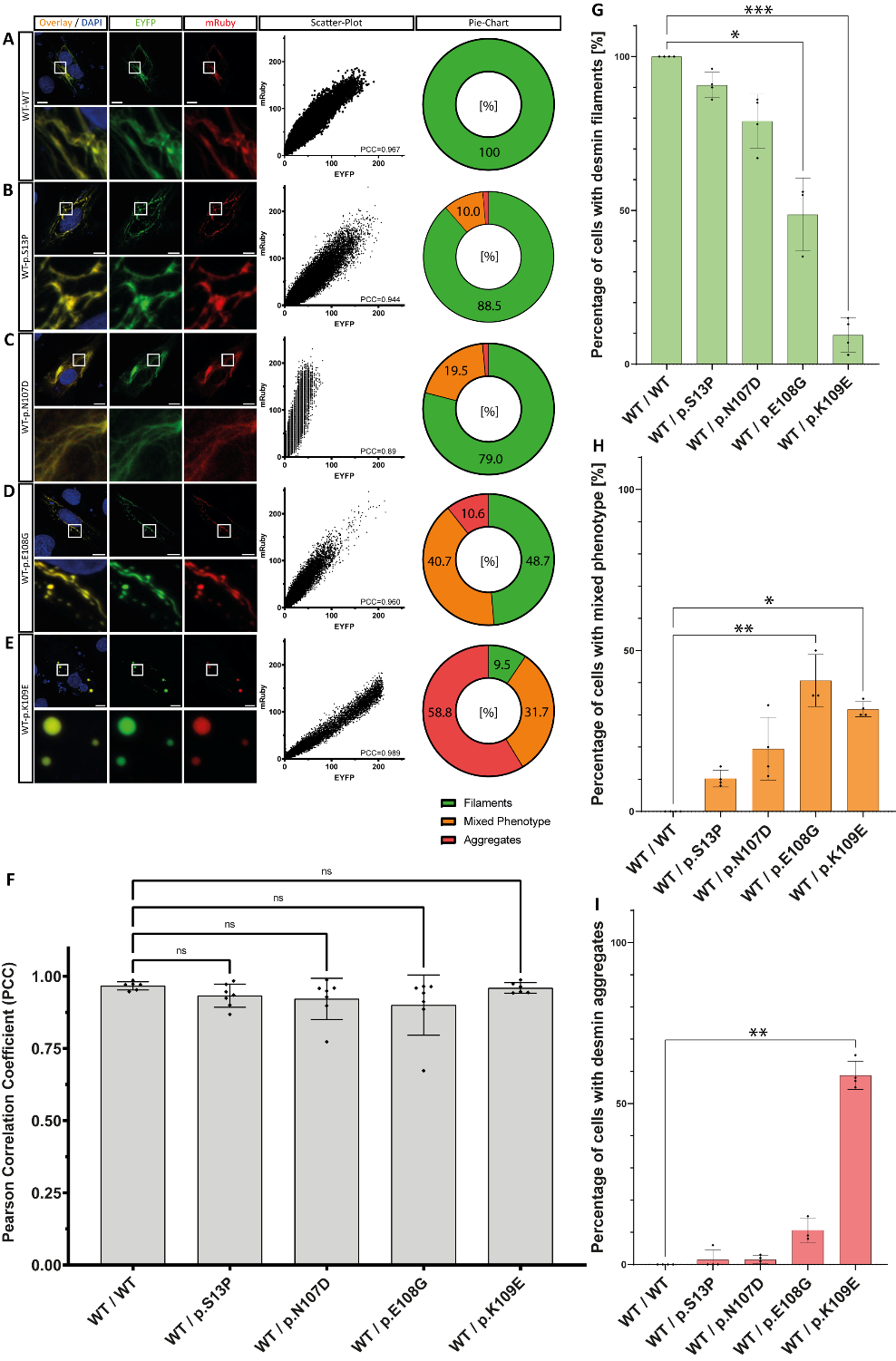


**Figure S10.** Co-expression experiments of mutant and wild-type desmin in H9c2 cells. **(A-E)** Representative images of double transfected H9c2 cells, expressing wild-type desmin (fused mRuby, red) and wild-type or mutant desmin (fused to EYFP, green). The overlap is shown in yellow. Nuclei were stained using DAPI and are shown in blue. Scale bars represent 20 µm. Representative scatter plots of the mRuby and EYFP channel used to determine the PCC are shown. **(F)** Statistical analysis of PCCs of the double transfected cells indicate a high colocalization of mutant and wild-type in filaments or aggregates. **(G-I)** Statistical analysis of desmin filament or aggregate formation using non-parametric Kruskal-Wallis test in double transfected H9c2 cells. All data are shown as mean ± standard deviation. *p≤0.05; **p≤0.01; ***p≤0.001; ****p≤0.0001.


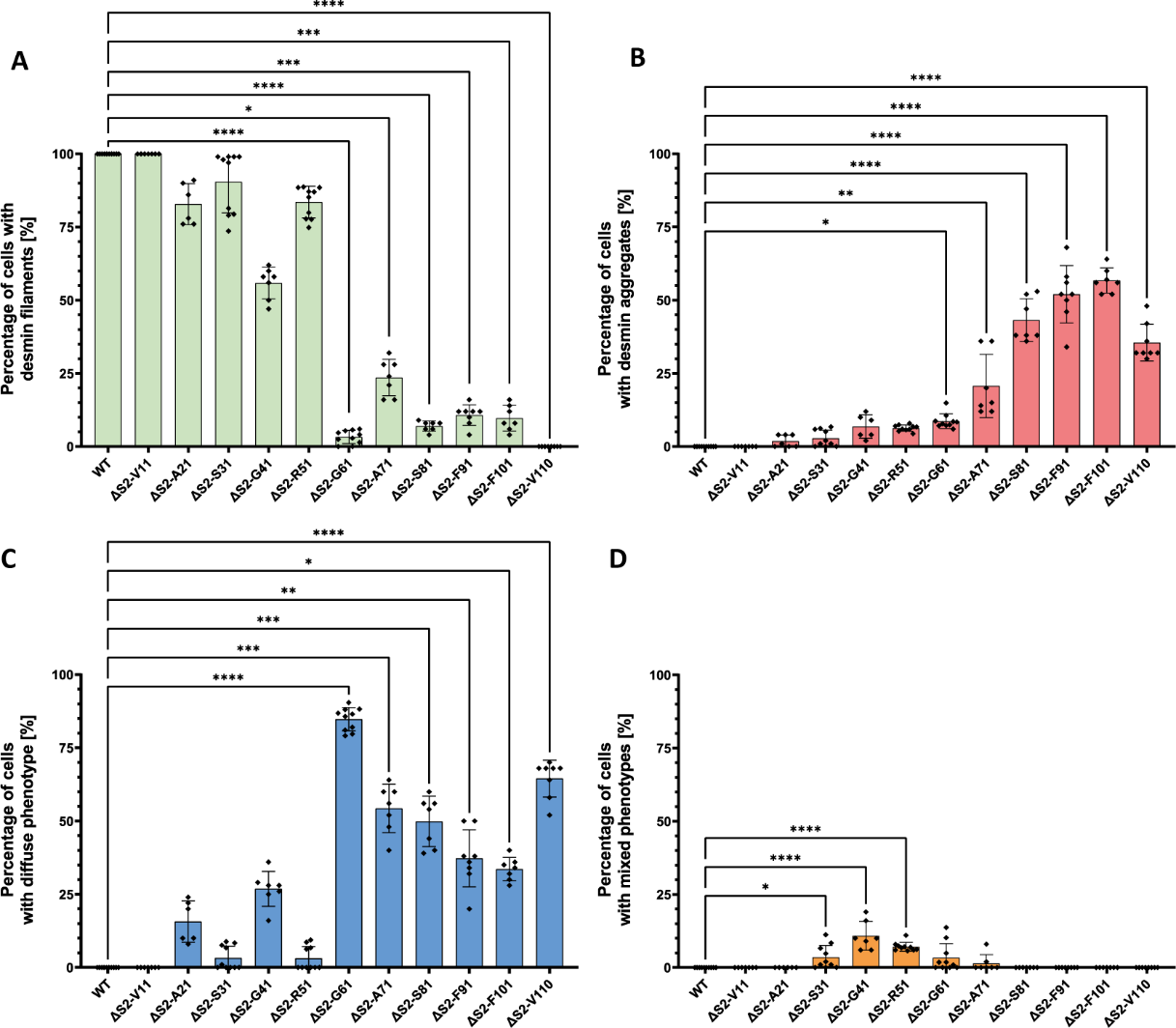


**Figure S11.** Filament formation of N-terminal desmin deletion mutants in H9c2 cells. **(A‑D)**Statistical analysis of desmin filament or aggregate formation was performed using non-parametric Kruskal-Wallis test in transfected H9c2 cells expressing wild-type desmin or desmin deletion mutants. All data are shown as mean ± standard deviation. *p≤0.05; **p≤0.01; ***p≤0.001; ****p≤0.0001.


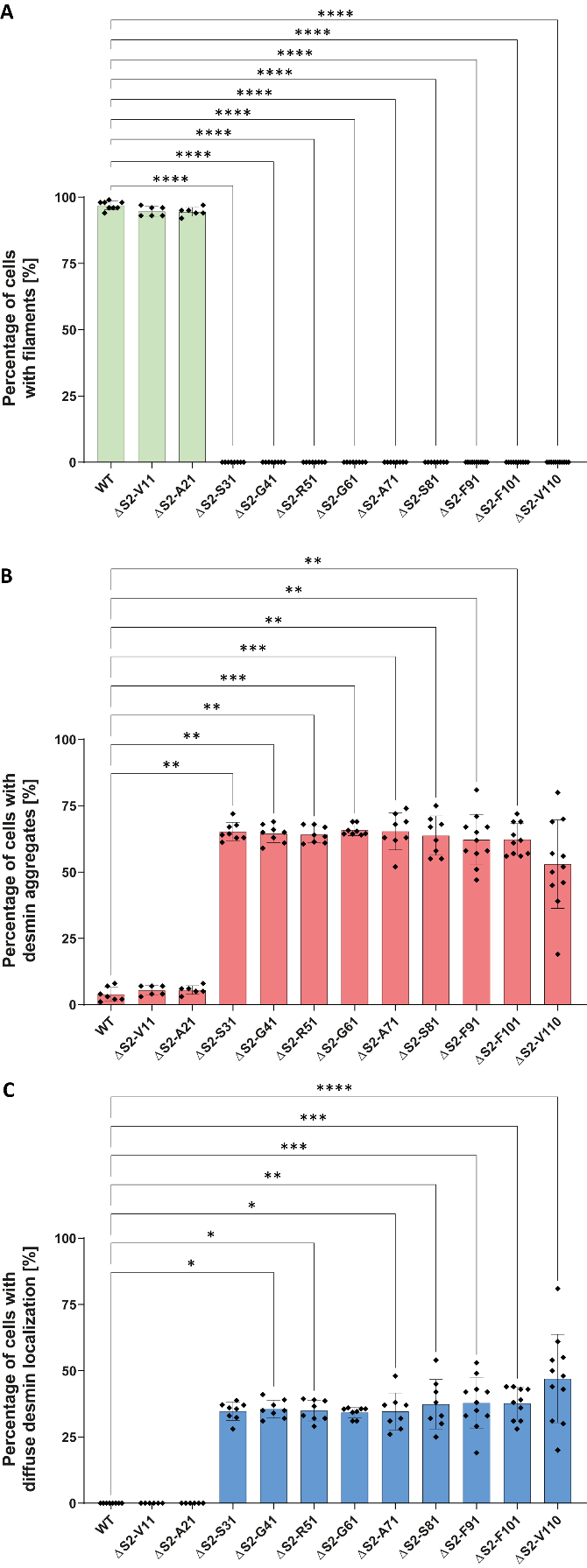


**Figure S12.** Filament formation of N-terminal desmin deletion mutants in SW-13 cells. **(A‑D)**Statistical analysis of desmin filament or aggregate formation was performed using non-parametric Kruskal-Wallis test in transfected SW-13 cells expressing wild-type desmin or desmin deletion mutants. All data are shown as mean ± standard deviation. *p≤0.05; **p≤0.01; ***p≤0.001; ****p≤0.0001.


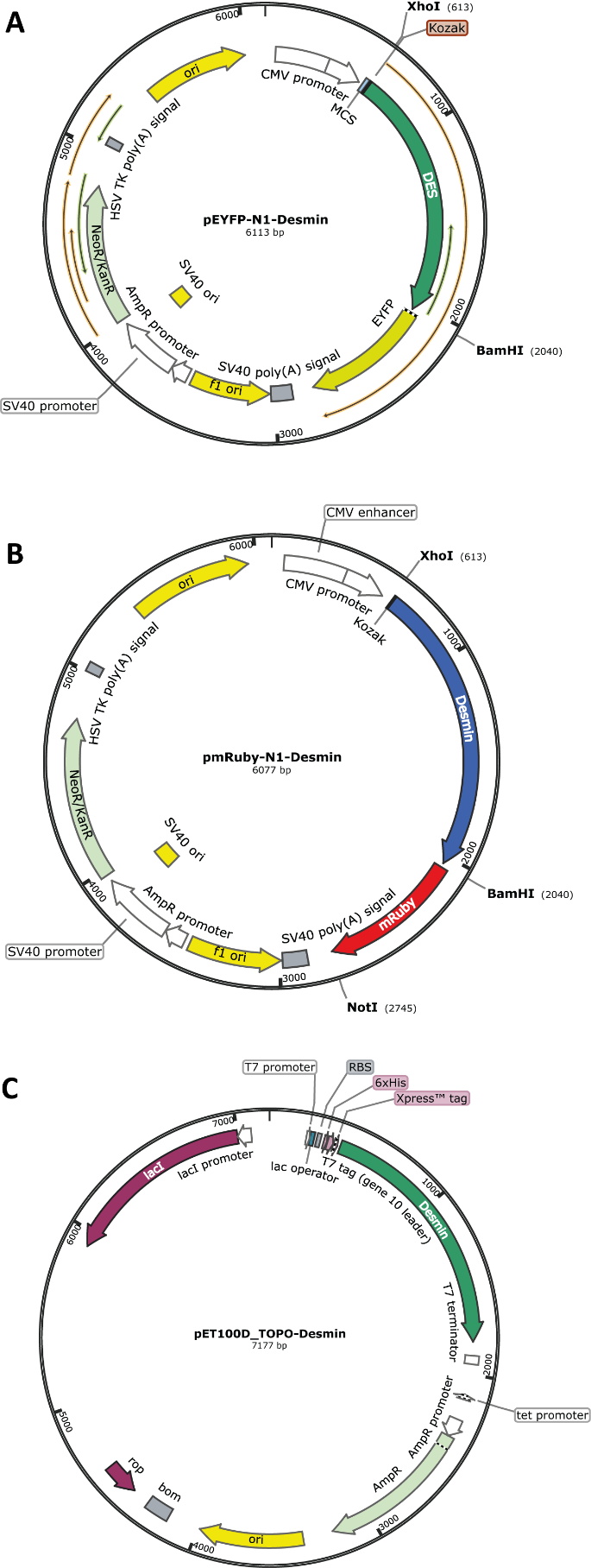


**Figure S13.** Schematic maps of the used plasmids. **(A)** pEYFP-N1-Desmin; **(B)** pmRuby-N1-Desmin and **(C)** pET100D-TOPO-Desmin.


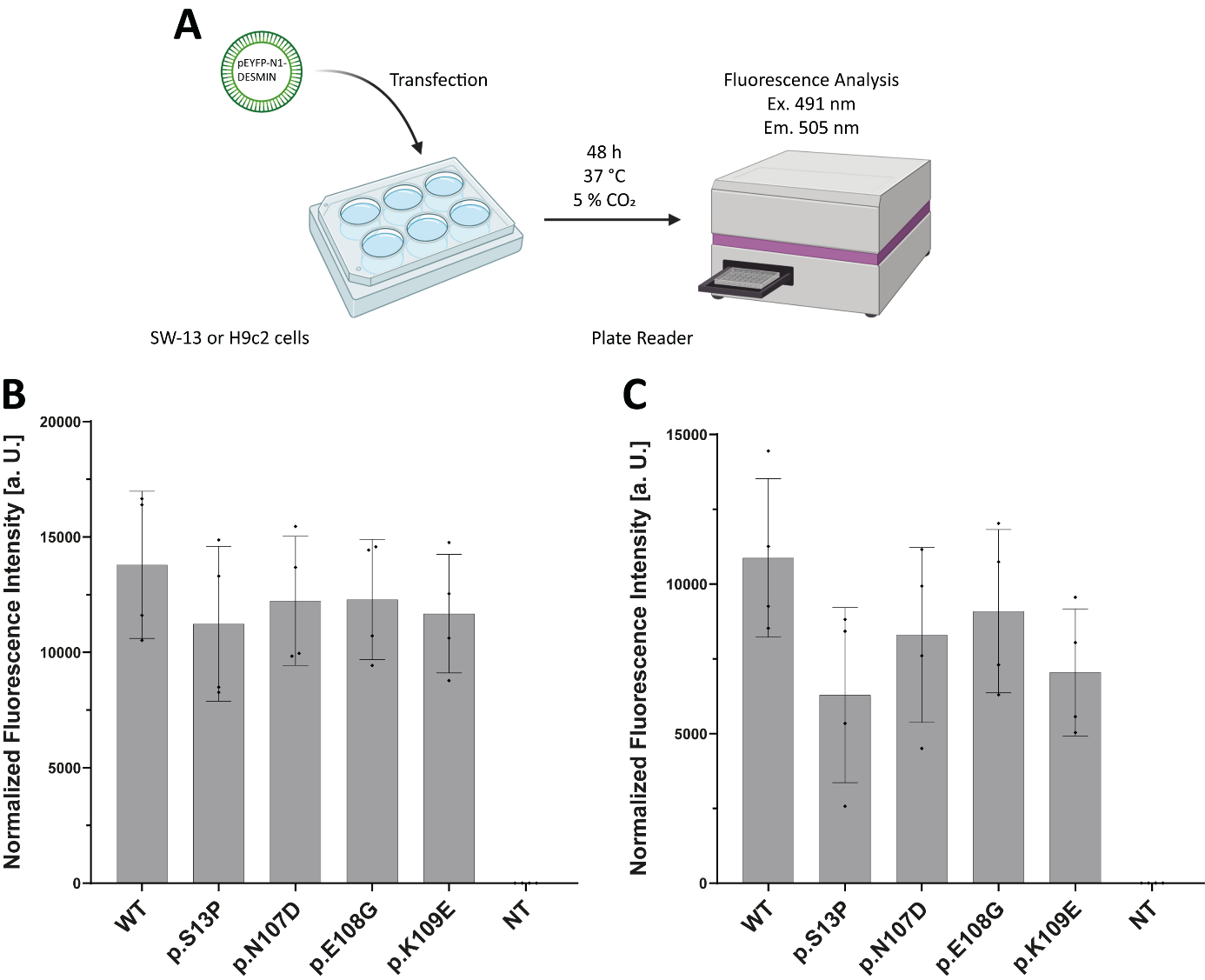


**Figure S14.** Expression analysis. **(A)** SW-13 and H9c2 cells were transfected with desmin constructs fused to EYFP. Fluorescence was analysed using a plate reader. Statistical analysis of the normalized fluorescence intensities revealed no obvious differences between wild-type and mutant desmin in **(B)** SW-13 and **(C)** H9c2 cells.


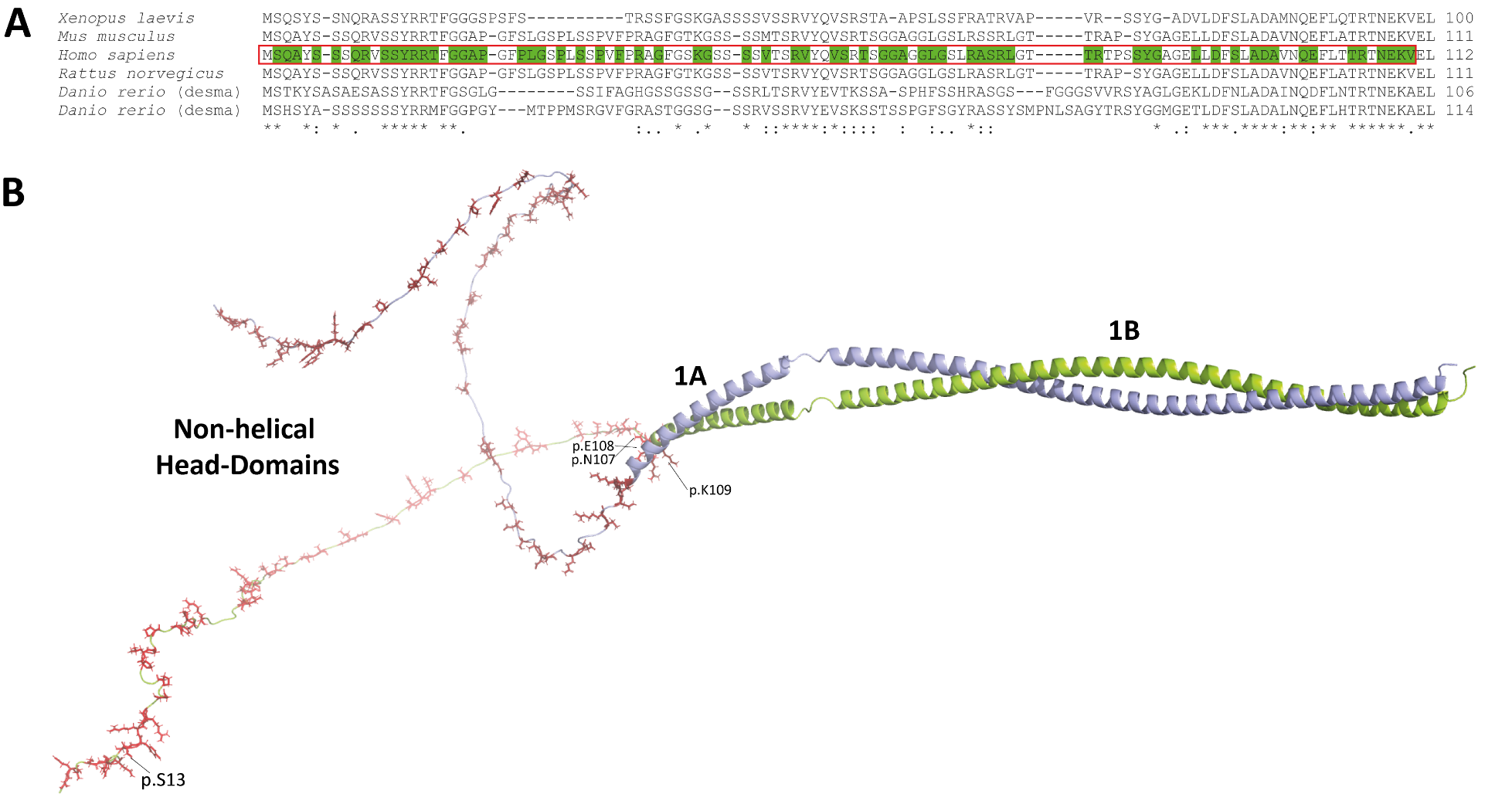


**Figure S15.** Schematic overview of the desmin head domain. **(A)** Partial sequence alignment of the desmin head domains (red box) from different species (*Xenopus laevis*, *Mus musculus*, *Homo sapiens*, *Rattus norvegicus* and *Danio rerio*). Positions of variants with unknown significance (VUS) are highlighted in green. Fully conserved amino acids are indicated with stars and conserved substitutions with colons. Dots mark conserved positions with weakly similar properties of these amino acids. **(B)** Structural overview of the non-helical head and the α-helical 1A and 1B domains of the desmin dimer modelled using AlphaFold. Amino acids affected by VUS are shown as red sticks.

**Table S1.** Overview about the used oligonucleotides.

| Name | Sequence 5‘-3‘ | Application |
| --- | --- | --- |
| DES_L110V_for | GCACCAACGAGAAG**T**TGGAGCTGCAGGAG | SDM |
| DES_L110V_rev | CTCCTGCAGCTCCA**A**CTTCTCGTTGGTGC | SDM |
| DES_K109E_for | GCGCACCAACGAG**G**AGGTGGAGCTGCA | SDM |
| DES_K109E_rev | TGCAGCTCCACCT**C**CTCGTTGGTGCGC | SDM |
| DES_E108G_for | ACGCGCACCAACG**G**GAAGGTGGAGCTG | SDM |
| DES_E108G_rev | CAGCTCCACCTTC**C**CGTTGGTGCGCGT | SDM |
| DES_N107D_for | GACCACGCGCACC**G**ACGAGAAGGTGGA | SDM |
| DES_N107D_rev | TCCACCTTCTCGT**C**GGTGCGCGTGGTC | SDM |
| DES_N107T_for | GACCACGCGCA**C**CACCGAGAAGGTGGA | SDM |
| DES_N107T_rev | TCCACCTTCTCG**G**TGGTGCGCGTGGTC | SDM |
| DES_R105L_for | GAGTTTCTGACCACGC**T**CACCAACGAGAAGGTG | SDM |
| DES_R105L_rev | CACCTTCTCGTTGGTG**A**GCGTGGTCAGAAACTC | SDM |
| DES_R105C_for | GAGTTTCTGACCACG**T**GCACCAACGAGAAGG | SDM |
| DES_R105C_rev | CCTTCTCGTTGGTGC**A**CGTGGTCAGAAACTC | SDM |
| DES_T104M_for | CCAGGAGTTTCTGACCA**T**GCGCACCAACG | SDM |
| DES_T104M_rev | CGTTGGTGCGC**A**TGGTCAGAAACTCCTGG | SDM |
| DES_T104R_for | CCAGGAGTTTCTGACCA**G**GCGCACCAACG | SDM |
| DES_T104R_rev | CGTTGGTGCGC**C**TGGTCAGAAACTCCTGG | SDM |
| DES_T104A_for | AGGAGTTTCTGACC**G**CGCGCACCAACGAG | SDM |
| DES_T104A_rev | CTCGTTGGTGCGCG**C**GGTCAGAAACTCCT | SDM |
| DES_E100A_for | CGCGGTGAACCAGG**C**GTTTCTGACCACGC | SDM |
| DES_E100A_rev | GCGTGGTCAGAAAC**G**CCTGGTTCACCGCG | SDM |
| DES_E100K_for | GACGCGGTGAACCAG**A**AGTTTCTGACCACGC | SDM |
| DES_E100K_rev | GCGTGGTCAGAAACT**T**CTGGTTCACCGCGTC | SDM |
| DES_Q99E_for | CCGACGCGGTGAAC**G**AGGAGTTTCTGACC | SDM |
| DES_Q99E_rev | GGTCAGAAACTCCT**C**GTTCACCGCGTCGG | SDM |
| DES_A96S_for | CTCACTGGCCGAC**T**CGGTGAACCAGGA | SDM |
| DES_A96S_rev | TCCTGGTTCACCG**A**GTCGGCCAGTGAG | SDM |
| DES_D95V_for | TTCTCACTGGCCG**T**CGCGGTGAACCAG | SDM |
| DES_D95V_rev | CTGGTTCACCGCG**A**CGGCCAGTGAGAA | SDM |
| DES_A94T_for | TGGACTTCTCACTG**A**CCGACGCGGTGAAC | SDM |
| DES_A94T_rev | GTTCACCGCGTCGG**T**CAGTGAGAAGTCCA | SDM |
| DES_S92L_for | GGCGAGCTGCTGGACTTCT**T**ACTGGCCGAC | SDM |
| DES_S92L_rev | GTCGGCCAGT**A**AGAAGTCCAGCAGCTCGCC | SDM |
| DES_D90V_for | GGCGAGCTGCTGG**T**CTTCTCACTGGCC | SDM |
| DES_D90V_rev | GGCCAGTGAGAAG**A**CCAGCAGCTCGCC | SDM |
| DES_D90A_for | GGCGAGCTGCTGG**C**CTTCTCACTGGCC | SDM |
| DES_D90A_rev | GGCCAGTGAGAAG**G**CCAGCAGCTCGCC | SDM |
| DES_D90H_for | AGGCGAGCTGCTG**C**ACTTCTCACTGGC | SDM |
| DES_D90H_rev | GCCAGTGAGAAGT**G**CAGCAGCTCGCCT | SDM |
| DES_L88R_for | GCGCAGGCGAGC**G**GCTGGACTTCTC | SDM |
| DES_L88R_rev | GAGAAGTCCAGC**C**GCTCGCCTGCGC | SDM |
| DES_G84S_for | CCCTCCTCCTAC**A**GCGCAGGCGAGC | SDM |
| DES_G84S_rev | GCTCGCCTGCGC**T**GTAGGAGGAGGG | SDM |
| DES_Y83H_for | CGCCCTCCTCC**C**ACGGCGCAGGC | SDM |
| DES_Y83H_rev | GCCTGCGCCGT**G**GGAGGAGGGCG | SDM |
| DES_S82F_for | GCACGCCCTCCT**T**CTACGGCGCAGG | SDM |
| DES_S82F_rev | CCTGCGCCGTAG**A**AGGAGGGCGTGC | SDM |
| DES_T77I_for | CCGGCTGGGGACCA**T**CCGCACGC | SDM |
| DES_T77I_rev | GCGTGCGG**A**TGGTCCCCAGCCGG | SDM |
| DES_T77A_for | GGCTGGGGACC**G**CCCGCACGCCC | SDM |
| DES_T77A_rev | GGGCGTGCGGG**C**GGTCCCCAGCC | SDM |
| DES_L74Q_for | GGCCAGCCGGC**A**GGGGACCACCC | SDM |
| DES_L74Q_rev | GGGTGGTCCCC**T**GCCGGCTGGCC | SDM |
| DES_R73L_for | GCGGGCCAGCC**T**GCTGGGGACCA | SDM |
| DES_R73L_rev | TGGTCCCCAGC**A**GGCTGGCCCGC | SDM |
| DES_R73Q_for | GCGGGCCAGCC**A**GCTGGGGACCA | SDM |
| DES_R73Q_rev | TGGTCCCCAGC**T**GGCTGGCCCGC | SDM |
| DES_S72R_for | CTGCGGGCCAG**G**CGGCTGGGGAC | SDM |
| DES_S72R_rev | GTCCCCAGCCG**C**CTGGCCCGCAG | SDM |
| DES_R70W_for | TGGGGTCGCTG**T**GGGCCAGCCGG | SDM |
| DES_R70W_rev | CCGGCTGGCCC**A**CAGCGACCCCA | SDM |
| DES_G67R_for | CCGGGGGCCTG**A**GGTCGCTGCGG | SDM |
| DES_G67R_rev | CCGCAGCGACC**T**CAGGCCCCCGG | SDM |
| DES_L66M_for | GGGCCGGGGGC**A**TGGGGTCGCTG | SDM |
| DES_L66M_rev | CAGCGACCCCA**T**GCCCCCGGCCC | SDM |
| DES_G65S_for | CGGGGCCGGG**A**GCCTGGGGTC | SDM |
| DES_G65S_rev | GACCCCAGGC**T**CCCGGCCCCG | SDM |
| DES_A63D_for | TCGGGCGGGG**A**CGGGGGCCTG | SDM |
| DES_A63D_rev | CAGGCCCCCG**T**CCCCGCCCGA | SDM |
| DES_G62R_for | CACGTCGGGC**A**GGGCCGGGGG | SDM |
| DES_G62R_rev | CCCCCGGCCC**T**GCCCGACGTG | SDM |
| DES_G61D_for | CGCACGTCGG**A**CGGGGCCGGG | SDM |
| DES_G61D_rev | CCCGGCCCCG**T**CCGACGTGCG | SDM |
| DES_T59M_for | GGTGTCGCGCA**T**GTCGGGCGGGG | SDM |
| DES_T59M_rev | CCCCGCCCGAC**A**TGCGCGACACC | SDM |
| DES_S57L_for | CCGCGTGTACCAGGTGT**T**GCGCACGTC | SDM |
| DES_S57L_rev | GACGTGCGC**A**ACACCTGGTACACGCGG | SDM |
| DES_V56L_for | CCGCGTGTACCAG**T**TGTCGCGCACGTC | SDM |
| DES_V56L_rev | GACGTGCGCGAC**A**ACTGGTACACGCGG | SDM |
| DES_V53L_for | GGTGACGTCCCGC**T**TGTACCAGGTGTC | SDM |
| DES_V53L_rev | GACACCTGGTAC**A**AGCGGGACGTCACC | SDM |
| DES_R52H_for | TCGGTGACGTCCC**A**CGTGTACCAGGTG | SDM |
| DES_R52H_rev | CACCTGGTACACG**T**GGGACGTCACCGA | SDM |
| DES_R52P_for | CGGTGACGTCCC**C**CGTGTACCAGGT | SDM |
| DES_R52P_rev | ACCTGGTACACG**G**GGGACGTCACCG | SDM |
| DES_R52S_for | CTCGGTGACGTCC**A**GCGTGTACCAGGT | SDM |
| DES_R52S_rev | ACCTGGTACACGC**T**GGACGTCACCGAG | SDM |
| DES_V49A_for | CTCCAGCTCGG**C**GACGTCCCGCG | SDM |
| DES_V49A_rev | CGCGGGACGTC**G**CCGAGCTGGAG | SDM |
| DES_S47I_for | AAGGGCTCCTCCA**T**CTCGGTGACGTCC | SDM |
| DES_S47I_rev | GGACGTCACCGAG**A**TGGAGGAGCCCTT | SDM |
| DES_G44V_for | TTTCGGCTCTAAGG**T**CTCCTCCAGCTCGG | SDM |
| DES_G44V_rev | CCGAGCTGGAGGAG**A**CCTTAGAGCCGAAA | SDM |
| DES_K43E_for | GGGCGGGTTTCGGCTCT**G**AGGGCTCCT | SDM |
| DES_K43E_rev | AGGAGCCCT**C**AGAGCCGAAACCCGCCC | SDM |
| DES_R37L_for | CCGTGTTCCCGC**T**GGCGGGTTTCG | SDM |
| DES_R37L_rev | CCGAAACCCGCC**A**GCGGGAACACGG | SDM |
| DES_R37W_for | CCCGTGTTCCCG**T**GGGCGGGTTTCG | SDM |
| DES_R37W_rev | CGAAACCCGCCC**A**CGGGAACACGGG | SDM |
| DES_R37G_for | CCGTGTTCCCG**G**GGGCGGGTTTC | SDM |
| DES_R37G_rev | GAAACCCGCCC**C**CGGGAACACGG | SDM |
| DES_F35L_for | GAGCTCGCCCGTGTT**A**CCGCGGGC | SDM |
| DES_F35L_rev | GCCCGCGG**T**AACACGGGCGAGCTC | SDM |
| DES_F35S_for | GCTCGCCCGTG**AG**CCCGCGGGCGG | SDM |
| DES_F35S_rev | CCGCCCGCGGG**CT**CACGGGCGAGC | SDM |
| DES_P33S_for | CCCGCTGAGCTCG**AG**CGTGTTCCCGCGG | SDM |
| DES_P33S_rev | CCGCGGGAACACG**CT**CGAGCTCAGCGGG | SDM |
| DES_S31R_for | CTCCCCGCTGAG**G**TCGCCCGTGTTC | SDM |
| DES_S31R_rev | GAACACGGGCGA**C**CTCAGCGGGGAG | SDM |
| DES_S31C_for | GCTCCCCGCTG**T**GCTCGCCCGTG | SDM |
| DES_S31C_rev | CACGGGCGAGC**A**CAGCGGGGAGC | SDM |
| DES_P29L_for | GCTCGGCTCCC**T**GCTGAGCTCGC | SDM |
| DES_P29L_rev | GCGAGCTCAGC**A**GGGAGCCGAGC | SDM |
| DES_G27D_for | GCTTCCCGCTCG**A**CTCCCCGCTGAG | SDM |
| DES_G27D_rev | CTCAGCGGGGAG**T**CGAGCGGGAAGC | SDM |
| DES_G27S_for | GCTTCCCGCTC**A**GCTCCCCGCTG | SDM |
| DES_G27S_rev | CAGCGGGGAGC**T**GAGCGGGAAGC | SDM |
| DES_G27R_for | GCTTCCCGCTC**C**GCTCCCCGCTG | SDM |
| DES_G27R_rev | CAGCGGGGAGC**G**GAGCGGGAAGC | SDM |
| DES_L26H_for | GGGCTTCCCGC**A**CGGCTCCCCGC | SDM |
| DES_L26H_rev | GCGGGGAGCCG**T**GCGGGAAGCCC | SDM |
| DES_P25L_for | CCCGGGCTTCC**T**GCTCGGCTCCC | SDM |
| DES_P25L_rev | GGGAGCCGAGC**A**GGAAGCCCGGG | SDM |
| DES_P22R_for | GGCGGGGCCC**G**GGGCTTCCCG | SDM |
| DES_P22R_rev | CGGGAAGCCC**C**GGGCCCCGCC | SDM |
| DES_G20E_for | CACCTTCGGCG**A**GGCCCCGGGCT | SDM |
| DES_G20E_rev | AGCCCGGGGCC**T**CGCCGAAGGTG | SDM |
| DES_G20R_for | GCACCTTCGGC**A**GGGCCCCGGGC | SDM |
| DES_G20R_rev | GCCCGGGGCCC**T**GCCGAAGGTGC | SDM |
| DES_G19R_for | CCGCACCTTC**C**GCGGGGCCCC | SDM |
| DES_G19R_rev | GGGGCCCCGC**G**GAAGGTGCGG | SDM |
| DES_T17N_for | CTACCGCCGCA**A**CTTCGGCGGGG | SDM |
| DES_T17N_rev | CCCCGCCGAAGT**T**GCGGCGGTAG | SDM |
| DES_T17S_for | CTACCGCCGCA**G**CTTCGGCGGGG | SDM |
| DES_T17S_rev | CCCCGCCGAAG**C**TGCGGCGGTAG | SDM |
| DES_R16S_for | TCCTCCTACCGC**A**GCACCTTCGGCG | SDM |
| DES_R16S_rev | CGCCGAAGGTGC**T**GCGGTAGGAGGA | SDM |
| DES_R15S_for | CGTGTCCTCCTAC**A**GCCGCACCTTCGG | SDM |
| DES_R15S_rev | CCGAAGGTGCGGC**T**GTAGGAGGACACG | SDM |
| DES_R15C_for | CGTGTCCTCCTAC**T**GCCGCACCTTCGG | SDM |
| DES_R15C_rev | CCGAAGGTGCGGC**A**GTAGGAGGACACG | SDM |
| DES_Y14H_for | GCGTGTCCTCCC**A**CCGCCGCACC | SDM |
| DES_Y14H_rev | GGTGCGGCGGT**G**GGAGGACACGC | SDM |
| DES_S13Y_for | CCAGCGCGTGTCCT**AT**TACCGCCGCACCTT | SDM |
| DES_S13Y_rev | AAGGTGCGGCGGTA**AT**AGGACACGCGCTGG | SDM |
| DES_S13P_for | AGCGCGTGTCC**C**CCTACCGCCGC | SDM |
| DES_S13P_rev | GCGGCGGTAGG**G**GGACACGCGCT | SDM |
| DES_S12F_for | GCCAGCGCGTGT**T**CTCCTACCGCCG | SDM |
| DES_S12F_rev | CGGCGGTAGGAG**A**ACACGCGCTGGC | SDM |
| DES_R10H_for | TCGTCCAGCCAGC**A**CGTGTCCTCCTAC | SDM |
| DES_R10H_rev | GTAGGAGGACACG**T**GCTGGCTGGACGA | SDM |
| DES_R10S_for | CTCGTCCAGCCAG**A**GCGTGTCCTCCTA | SDM |
| DES_R10S_rev | TAGGAGGACACGC**T**CTGGCTGGACGAG | SDM |
| DES_R10C_for | CTCGTCCAGCCAG**T**GCGTGTCCTCCTA | SDM |
| DES_R10C_rev | TAGGAGGACACGC**A**CTGGCTGGACGAG | SDM |
| DES_Q9E_for | CTACTCGTCCAGC**G**AGCGCGTGTCCTC | SDM |
| DES_Q9E_rev | GAGGACACGCGCT**C**GCTGGACGAGTAG | SDM |
| DES_S7F_for | CAGGCCTACTCGT**T**CAGCCAGCGCGTG | SDM |
| DES_S7F_rev | CACGCGCTGGCTG**A**ACGAGTAGGCCTG | SDM |
| DES_S6L_for | GCCAGGCCTACT**T**GTCCAGCCAGCG | SDM |
| DES_S6L_rev | CGCTGGCTGGAC**A**AGTAGGCCTGGC | SDM |
| DES_S6W_for | GCCAGGCCTACT**G**GTCCAGCCAGCG | SDM |
| DES_S6W_rev | CGCTGGCTGGAC**C**AGTAGGCCTGGC | SDM |
| DES_A4T_for | TCACCATGAGCCAG**A**CCTACTCGTCCAGC | SDM |
| DES_A4T_rev | GCTGGACGAGTAGGTC**T**GGCTCATGGTGA | SDM |
| DES_Q3R_for | GTCACCATGAGCC**G**GGCCTACTCGTCC | SDM |
| DES_Q3R_rev | GGACGAGTAGG**C**CCGGCTCATGGTGAC | SDM |
| DES_S2I_for | CGAGGCCGTCACCATGA**T**CCAGGCCTA | SDM |
| DES_S2I_rev | TAGGCCTGG**A**TCATGGTGACGGCCTCG | SDM |
| CMV_for | CGCAAATGGGCGGTAGGCGTG | Sanger Sequencing |
| EGFP_N_rev | GCTTGCCGTAGGTGGCATC | Sanger Sequencing |
| T7_for | TAATACGACTCACTATAGGG | Sanger Sequencing |
| T7_rev | GCTAGTTATTGCTCAGCGGT | Sanger Sequencing |
| DES_HEAD_del_for | GGCCGTCACCATGGAGCTGCAGGAGC | SDM |
| DES_HEAD_del_rev | GCTCCTGCAGCTCCATGGTGACGGCC | SDM |
| DES-S2-V11del_for | GGCCGTCACCATGTCCTCCTACCGCC | SDM |
| DES-S2-V11del_rev | GGCGGTAGGAGGACATGGTGACGGCC | SDM |
| DES-S2-A21del_for | CGGGAAGCCCGGCATGGTGACGGC | SDM |
| DES-S2-A21del_rev | GCCGTCACCATGCCGGGCTTCCCG | SDM |
| DES-S2-G41del_for | GGAGGAGCCCTTAGACATGGTGACGGCCTC | SDM |
| DES-S2-G41del_rev | GAGGCCGTCACCATGTCTAAGGGCTCCTCC | SDM |
| DES-S2-A71del_for | CCCCAGCCGGCTCATGGTGACGGC | SDM |
| DES-S2-A71del_rev | GCCGTCACCATGAGCCGGCTGGGG | SDM |
| DES-S2-S81del_for | CTGCGCCGTAGGACATGGTGACGGCC | SDM |
| DES-S2-S81del_rev | GGCCGTCACCATGTCCTACGGCGCAG | SDM |
| DES-S2-F91del_for | CGTCGGCCAGTGACATGGTGACGGCC | SDM |
| DES-S2-F91del_rev | GGCCGTCACCATGTCACTGGCCGACG | SDM |
| DES-S2-F101del_for | TGCGCGTGGTCAGCATGGTGACGGCC | SDM |
| DES-S2-F101del_rev | GGCCGTCACCATGCTGACCACGCGCA | SDM |
| XhoI_Kozak_ATG_DES_S31del_for | TCAGATCTCGAGGCCGTCACCATGTCGCCCGTGTTCCCGCGG | PCR / Cloning |
| XhoI_Kozak_ATG_DES_S51del_for | TCAGATCTCGAGGCCGTCACCATGCGCGTGTACCAGGTGTCGC | PCR / Cloning |
| XhoI_Kozak_ATG_DES_G61_for | TCAGATCTCGAGGCCGTCACCATGGGGGCCGGGGGCCTGGGG | PCR / Cloning |
| BamHI_DES_rev | ACCGGTGGATCCCCGAGCACTTCATGCTGCTGCTGTG | PCR / Cloning |

PCR=Polymerase Chain Reaction; SDM=Site-Directed-Mutagenesis.
